# Insight on Mutation-Induced Resistance from Molecular Dynamics Simulations of the Native and Mutated CSF-1R and KIT

**DOI:** 10.1371/journal.pone.0160165

**Published:** 2016-07-28

**Authors:** Priscila Da Silva Figueiredo Celestino Gomes, Isaure Chauvot De Beauchêne, Nicolas Panel, Sophie Lopez, Paulo De Sepulveda, Pedro Geraldo Pascutti, Eric Solary, Luba Tchertanov

**Affiliations:** 1 Laboratoire de Biologie et Pharmacologie Appliquée (LBPA), ENS Cachan, CNRS, Université, Paris-Saclay, 94235 Cachan, France; 2 Laboratório de Modelagem e Dinâmica Molecular, Instituto de Biofísica Carlos Chagas FilhoUniversidade Federal do Rio de Janeiro, 373 av. Carlos Chagas Filho, 21941-902, Rio de Janeiro, Brazil; 3 INSERM U1068, Centre de Recherche en Cancérologie de Marseille, 13009 Marseille, France; 4 Inserm UMR1170, Gustave Roussy, 114 rue Edouard Vaillant, 94805 Villejuif cedex, France; 5 Paris-Sud University, Faculty of Medicine, Le Kremlin-Bicêtre, France; 6 Centre de Mathématiques et de Leurs Applications, (CMLA), ENS Cachan, CNRS, Université, Paris-Saclay, 94235 Cachan, France; University of Minnesota, UNITED STATES

## Abstract

The receptors tyrosine kinases (RTKs) for the colony stimulating factor-1, CSF-1R, and for the stem cell factor, SCFR or KIT, are important mediators of signal transduction. The abnormal function of these receptors, promoted by gain-of-function mutations, leads to their constitutive activation, associated with cancer or other proliferative diseases. A secondary effect of the mutations is the alteration of receptors’ sensitivity to tyrosine kinase inhibitors, compromising effectiveness of these molecules in clinical treatment. In particular, the mutation V560G in KIT increases its sensitivity to Imatinib, while the D816V in KIT, and D802V in CSF-1R, triggers resistance to the drug. We analyzed the Imatinib binding affinity to the native and mutated KIT (mutations V560G, S628N and D816V) and CSF-1R (mutation D802V) by using molecular dynamics simulations and energy calculations of Imatinib•target complexes. Further, we evaluated the sensitivity of the studied KIT receptors to Imatinib by measuring the inhibition of KIT phosphorylation. Our study showed that (i) the binding free energy of Imatinib to the targets is highly correlated with their experimentally measured sensitivity; (ii) the electrostatic interactions are a decisive factor affecting the binding energy; (iii) the most deleterious impact to the Imatinib sensitivity is promoted by D802V (CSF-1R) and D816V (KIT) mutations; (iv) the role of the juxtamembrane region, JMR, in the imatinib binding is accessory. These findings contribute to a better description of the mutation-induced effects alternating the targets sensitivity to Imatinib.

## Introduction

**Fig 1:**
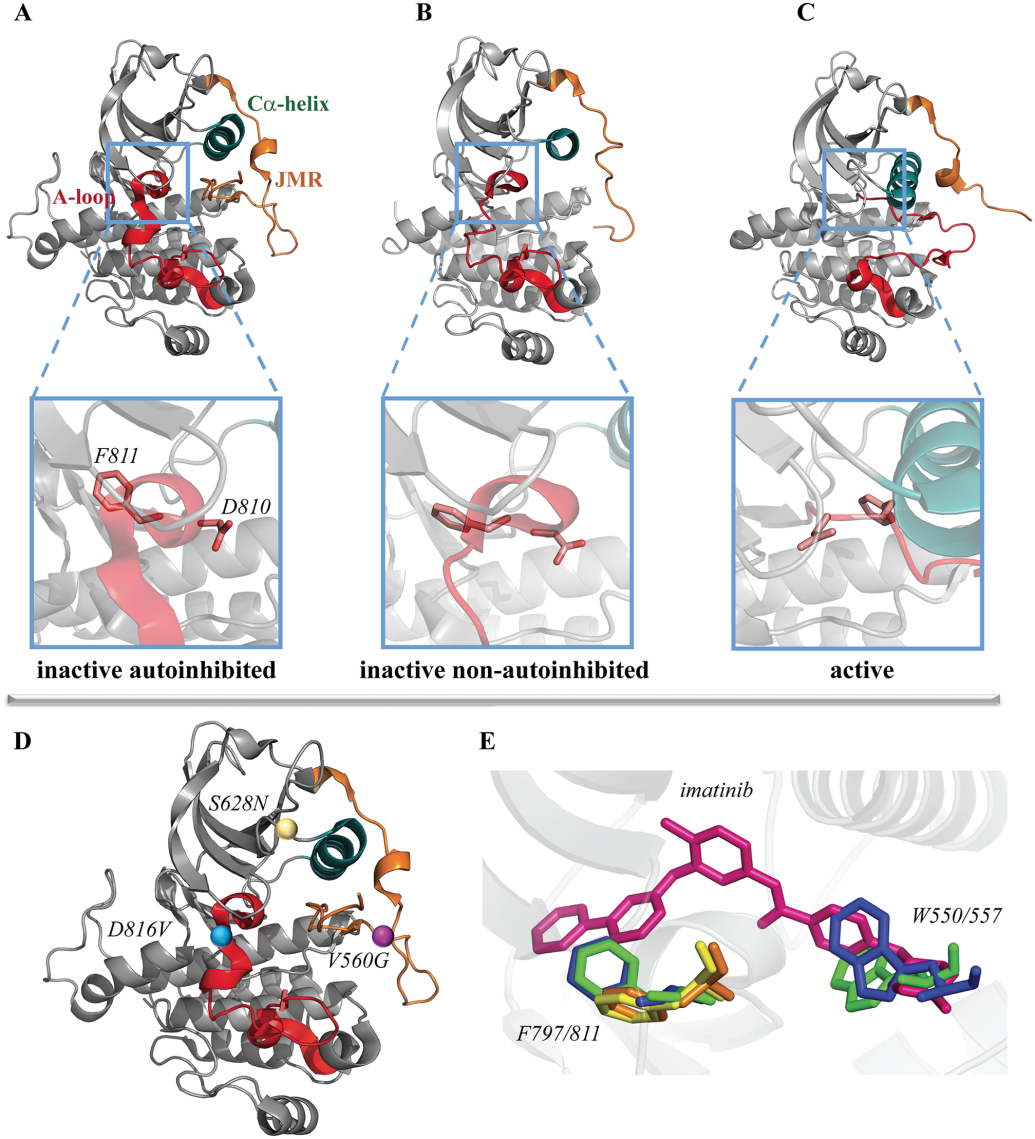
Structure of cytoplasmic region in RTKs. **(A-C)** Crystallographic structures (referenced with pdb code) of KIT in the inactive autoinhibited (1T45, left), inactive non-autoinhibited (1T46, middle) and active (1PKG, right) states are presented as cartoon. Zoomed views (*bottom panel*) show the active site of receptor with DFG motif (D810-F811-G812) represented in sticks. **(D)** Point mutations D816, V560 and S628 are showed in KIT (inactive state) as balls. In (**A-D**) the key structural fragments are highlighted in color—the αC-helix is in green, the activation loop (A-loop) is in red, the juxtamembrane region (JMR) is in orange. (**E**) Superposition of the Imatinib-binding site in structures of KIT (green) and CSF-1R (blue) in their auto-inhibited state non-bound to imatinib (PDB codes: 1T45 and 2OGV, respectively) and the structures of KIT (orange) and CSF-1R (yellow) bound to imatinib (1T46 and 4R7I, respectively). While a part of the JMR is absent in the crystallographic data, the residue W550/557 is not shown in the structures bound to imatinib.

Receptors tyrosine kinases (RTKs) act as primary mediators of the ligand-induced responses to control cellular signaling. The type III RTKs, comprising the stem cell factor (SCF) receptor KIT, the macrophage colony-stimulating factor-1 (CSF-1) receptor CSF-1R (or FMS), the platelet-derived growth factor α and β (PDGFR-α and PDGFR-β) and the FMS-like tyrosine kinase 3 (FLT3) receptors, are crucial for the development and physiology of different cells under normal conditions, and are implicated in different diseases [1]. The ligand-induced dimerization of the extracellular domain, leading to activation of the intracellular tyrosine kinase domain (TKD), promotes a conformational switch of key regulatory elements—the activation (A-) loop, the Cα-helix and the juxtamembrane region (JMR)–from inactive to active state (Fig 1A–1C) required for the phosphotransfer reaction [2]. Phosphorylation of specific tyrosine residues in RTKs controls various inter- and intra-cellular signaling pathways. The kinase activity and post-transduction processes are highly ordered and tightly regulated in normal cells [3]. Their constitutive activation promoted by mutations is associated with different forms of cancer [3–6]. Conversely, constitutive loss of function CSF-1R mutations were recently involved in severe neurodegenerative disorders [7]. RTKs are therefore crucial objects for fundamental research in biology and important targets for drug development.

The high sequence similarity and structural conservation of the kinase ATP-binding pocket—the site targeted by most kinase inhibitors—is the main challenge in the development of selective inhibitors of tyrosine kinase activity. Nevertheless, many of such ATP competitive inhibitors have been conceived and are currently used in the clinic or are under clinical trials. Debates on development of either selective or multi-targets inhibitors are still ongoing. On the one side, the use of a selective inhibitor of a particular kinase for a given tumor diminishes the possible non-desirable side effects. On the other side, a multi-targeted inhibitor inhibits divers signal transduction pathways that coexist in an apt tumor, thus providing a higher therapeutic efficacy. The archetype of such multi-kinase inhibitors is Imatinib (marketed by Novartis as *Gleevec* or *Glivec*), a rationally designed signal transduction inhibitor (antineoplastic agent), that selectively binds to a limited number of targets (KIT, BCR/ABL, RET proto-oncogene, CSF-1R, PDGFR-α and PDGFR-β) [8] and acts either as inhibitor (BCR/ABL, RET) or antagonist (KIT, CSF-1R, PDGFR-α and PDGFR-β) (DrugBank, http://www.drugbank.ca/).

Imatinib is successfully used for the treatment of several malignancies, such as Philadelphia chromosome-positive chronic myelogenous leukaemia (CML) [9], acute lymphoblastic leukaemia (ALL) [10] and KIT-positive gastrointestinal stromal tumors (GISTs) [11]. However, after several years of clinical use, it becomes clear that Imatinib-based treatment constantly results in acquired resistance coming from appearances of point mutations in the target kinase. Those mutations usually occur within amino-acid sequences that encode crucial structural (and functional) elements of the kinase, such as the ‘gatekeeper’ residues [12–14], the P-loop [15–17], the JMR and the A-loop amino acids [18,19]. In fact, therapeutic inactivation of protein creates selective pressures for tumor cells, analogous to those in natural selection, to evolve towards resistance phenomenon, mainly through the emergence of minor preexisting resistant clones [20,21]. The molecular mechanisms of the target resistance to Imatinib are not yet fully understood. The description of such mechanisms at the atomic scale will lead to the straightforward development of new generation of tyrosine kinase inhibitors, effective against the mutated targets.

Despite of numerous attempts, the structural-based explaining of Imatinib selectivity by the targets is only partial [22–25]. Extensive computational studies shed light on different aspects of Imatinib recognition by the native targets [26–31]. In particular, molecular dynamics (MD) simulations and free energy calculations on kinase-Imatinib complexes indicated comparable binding free energies of Imatinib to different kinases—Abl, KIT, Lck and Src [26]. Computing the binding free energy of Imatinib to Abl with the hybrid method QM-MM/PBSA differentiated the forces contributing to the inhibitor binding and showed that the van der Waals energy was the main component favoring the binding of a non-protonated imatinib [32]. Another detailed computational study applying FEP/MD simulations and umbrella sampling [33] showed that the selectivity of Imatinib to Abl over Src is a consequence of distinct stabilities of the targeted conformation of the kinase domain in the two receptors, which apparently affects the conformational selection by the drug [27]. A similar conclusion was delivered for the Imatinib binding by the wild-type targets KIT and Lck [27].

The mutation effects on the energy of Imatinib binding to the kinases were computationally studied for Bcr-Abl [29,34–36], FGFR [37,38] and KIT [39]. Exploring the X-ray structure of Imatinib—KIT complex by diverse online servers lead to the prediction of the impact of KIT mutations on the stability and dynamics of the non-bound target and to the evaluation of the role of particular residues to Imatinib binding [39].

We have previously proposed mechanisms of the mutation-induced constitutive activation of two RTKs, KIT and CSF-1R, having the equivalent D816V/D802V mutation [40,41]. These two mutants are highly resistant to Imatinib [18,42]. We have shown that the structural and dynamical effects induced by the KIT oncogenic mutations V506D/G, D816V/Y/N/H and S628N (Fig 1D), correlate with the auto-activation rates of KIT mutants measured *in vivo* and *in vitro* and their Imatinib sensitivity [43,44].

To examine rigorously the mutation-induced effects on Imatinib affinity towards these RTKs, we studied the structural, dynamical and thermodynamic properties of molecular complexes formed by their clinically relevant mutants and Imatinib. Two RTKs, KIT and CSF-1R, each in the native and mutated states, with the point mutations either alternating sensitivity to Imatinib or not, were considered as targets. As in our previous studies of non-bound KIT and CSF-1R, we used all-atom MD simulations and binding free energy (MM/PBSA) calculation. We aimed to distinguish between the binding affinities of Imatinib in the different complexes and to identify the main factors driving the binding of Imatinib to the native and mutated targets. Our study demonstrated that the Imatinib binding energy to the native and mutated KIT and CSF-1R targets is quite different. The electrostatic interactions between the protonated Imatinib and the targets residues were identified as a main factor contributing to such difference, and their alternation leads either to Imatinib resistance or to increasing of the target sensitivity to this inhibitor. Further, we experimentally evaluated the relative sensitivity of the various KIT receptors to Imatinib by the inhibition of KIT phosphorylation. The two types of data, obtained *in silico* and measured *in vitro*, were found to be highly correlated, strongly suggesting that the chosen computational methods are satisfying to produce biologically interpretable results.

## Materials and Methods

### Targets selection

Structural models of the inactive state of the cytoplasmic domain of CSF-1R and KIT in their native or wild-type (WT) (CSF-1R^WT^ and KIT^WT^) and mutated forms (CSF-1R^D802V^, KIT^V560G^, KIT^S628N^ and KIT^D816V^) were selected from our previous MD simulations [40–44]. Representative conformations over the MD trajectories were extracted by a fast clustering, based on convergence analysis [45]. Briefly, a first *reference* structure is initially picked up randomly and associated with a bin of MD conformations distant by less than an arbitrary RMSD cut-off (here we chose 2.0 Å); then other r*eference* structures are picked up randomly among the remaining MD conformations, in an iterative way until no MD conformations are remaining. Then each MD conformation in the total pool is associated to its closest (RMSD) *reference* structure, to form as many *reference* clusters. Considering only the Cα atoms, we applied this technique to a concatenated trajectory of KIT, containing two MD replicas of 50 ns for each form of the protein (KIT^WT^, KIT^V560G^, KIT^S628N^ and KIT^D816V^), and a concatenated trajectory of CSF-1R, containing two replicas of 50 ns for each form of the protein (CSF-1R^WT^ and CSF-1R^D802V^). Each MD replica contained 5 000 frames, but 500 frames generated during the first 5 ns were discarded. The total number of conformations/frames are 36 000 for KIT and 18 000 for CSF-1R. Each concatenated trajectory was spliced into the ‘*sections*’ describing each form of the receptor. Generally, a good convergence is reached when each *reference structure* is represented by conformations from all the parts of MD trajectories. In our case, instead of taking this well-represented *reference structures*, we have chosen the ones that were more represented for a particular target, instead of been visited by all forms, WT or mutants, of CSF-1R and KIT. Only one structure for each form of KIT or CSF-1R was selected for further calculations. Since the N-terminal portion of the JMR in these selected structures is buried into the ATP-binding site provoking a clash with the inhibitor, the JMR residues 543–581 in CSF-1R and 547–588 in KIT were manually removed from each selected structure, except for KIT^V560G^ in which the JMR was truncated at position 558 to conserve the mutated residue. Further, we have prepared the other KIT targets (KIT^WT^, KIT^S628N^ and KIT^D816V^) with the JMR, truncated as in KIT^V560G^.

### Molecular docking

The preparation of the receptors and imatinib structures, as well as the docking runs, were performed using the Schrödinger suite Maestro (Schrödinger Release 2014). The module *protein preparation wizard* was used to re-assign hydrogens, charges and to minimize the structures of WT CSF-1R/KIT and mutants, using the default parameters. Imatinib coordinates were retrieved from the crystallographic structure of KIT in complex with this inhibitor– 1T46 [25]. Imatinib was prepared using *LigPrep* (Schrödinger Release 2014) in the environment at pH 7.0. From the possible protonation states of Imatinib, generated by *LigPrep*, we have chosen the protonated state (+1), which seems to be the correct state for this inhibitor in complex with kinases, according to its predicted theoretical value [26]. The receptor structures were superposed to the crystal structure 1T46 to center the docking workspace on the ligand. The docking calculations were performed by using the *Induced Fit Docking* (IFD) [46] with the *Extended Sampling* protocol in which all targets residues positioned at a distance inferior to 5 Å from Imatinib were considered flexible. The binding affinity of Imatinib to the targets was characterized by docking scores (GLIDE and IFD). Each docking model was superposed with the crystal structure of the Imatinib-bound KIT (1T46) using *Maestro* from the Schrödinger suite, and the RMSD values were calculated in respect to 1T46. The best generated models of Imatinib•target complexes (target is CSF-1R^WT^, CSF-1R^D802V^, KIT^WT^, KIT^V560G^, KIT^S628N^ and KIT^D816V^ with the entirely truncated JMR), reconstituting the Imatinib position in 1T46, were used for MD simulations. Imatinib was manually placed into KIT^WT^, KIT^S628N^ and KIT^D816V^ structures bearing the partially truncated JMR. This was easily done by superposition with the successfully docked complexes for their truncated forms. Possible steric clashes were eliminated by energy minimization of the complexes.

### Molecular dynamics simulations

#### Imatinib preparation

To generate the Imatinib topology parameters compatible with CHARMM all-atoms force field [47], an Imatinib structure was retrieved from the Zinc Database (http://zinc.docking.org/) and used as input for the web server Swissparam [48].

#### Set up of the systems

The set-up of MD simulations was performed with the CHARMM27 all-atom force field integrated in GROMACS package 4.6.5 [49]. For simulations, each model was placed in a cubic box and centred with a 1.2 nm distance to the faces under periodic boundary conditions and solvated with explicit TIP3P water molecules [50]. Cl^-^ counter ions were added when necessary to neutralize the overall charge. The minimization procedure consisted of two steps: (i) steepest descent energy minimization (EM) with the solute atoms restrained and (ii) EM with all atoms free. The equilibration procedure was performed on the solvent, keeping the solute atoms (except H-atoms) restrained for 500 ps at 310 K and a constant volume (canonical NVT ensemble).

#### Production of MD trajectories

Two runs of 50-ns MD simulations were carried out for each complex ‘Imatinib•target’ (target is CSF-1R^WT^, CSF-1R^D802V^, KIT^WT^, KIT^V560G^, KIT^S628N^ and KIT^D816V^ with the entirely and partially cleaved JMR) and for each cleaved wild-type target in absence of Imatinib, further called ‘non-bound’ or ‘apo’ form. The temperature of solute (Imatinib•target) and solvent (water and ions) was separately coupled to the velocity rescale thermostat [51] at 310 K with relaxation time of 0.1 ps. The pressure was maintained at 1 atm by isotropic coordinate scaling (NPT ensemble) with relaxation time of 1 ps using Berendsen thermostat [52]. A time step of 2 fs was used to integrate the equations of motion based on the Leap-Frog algorithm [53]. The Lennard-Jones interactions were shifted to a cut-off 1.4 nm, and the Particle Mesh Ewald (PME) method [54] was used to treat long-range electrostatic interactions. The list for the electrostatic interactions was updated every 5 steps. All bonds were controlled using the Linear Constraint Solver (P-LINCS) algorithm [55]. The SETTLE algorithm [56] was used to constrain the water molecules geometry. Coordinates files were recorded every 10 ps.

#### Analysis of the trajectories

The MD trajectories were analysed (RMSDs and RMSFs computation and Principle Component Analysis) with tools included in the GROMACS package. The first 5 ns of each trajectory (equilibration time), were removed *prior* to analysis. A convergence analysis was performed using an ensemble-based approach described in details in [40–41,44]. Each analysis was performed on 9 000 conformations generated over two MD runs of 45 ns per simulation. Visual inspection of the MD data was made with PyMOL [57] and VMD [58]. Graphs were generated using Grace (http://plasma-gate.weizmann.ac.il/Grace/).

### Free energy calculation

Standard MM-PBSA (Molecular Mechanics—Poisson Boltzmann Surface Area) method [59] has been used for calculation of thermodynamic parameters and free energy of binding. In MM-PBSA, the free energy difference of binding (Δ*G*_*bind*_) between a ligand (L is Imatinib) and a receptors (R) to form a complex RL is defined as:

$$\Delta G_{bind}=\Delta H-T\Delta S≅\Delta E_{MM}+\Delta G_{sol}-T\Delta S$$ (1)

$$\Delta E_{MM}=\Delta E_{int}+\Delta E_{elect}+\Delta E_{vdw}$$ (2)

where Δ*E*_*MM*_, Δ*G*_*sol*_ and −TΔS are the changes of the gas phase MM energy, the solvation free energy and the conformational entropy upon binding, respectively.

Δ*E*_*MM*_ includes Δ*E*_*int*_ (covalent bonding including the bond, angle and dihedral energies), electrostatic Δ*E*_*elect*_ and van der Waals Δ*E*_*vdw*_ energies. Δ*G*_*sol*_ is the sum of the electrostatic solvation energy (polar contribution) and non-electrostatic solvation component (non-polar contribution). The polar contribution was calculated using PB model, while the non-polar free energy estimated by solvent accessible surface area (SASA).

Δ*G*_*bind*_ was computed for each generated Imatinib•target complex over the merged trajectories containing 9 000 frames (and also for the individual MD simulation replicas over a trajectory containing 4500 frames). We used the *g_mmpbsa* module of GROMACS, which combines subroutines from GROMACS and APBS [60] to calculate the enthalpy contribution (ΔH) to the Gibbs free energy and the per-residue energy decomposition. The energy components Δ*E*_*MM*_ and the polar and non-polar components of Δ*G*_*sol*_ were calculated in the bound and unbound form, and subsequently their contribution to the binding energy $\Delta R_{x}^{BE}$ of residue *x* [61] was calculated as follows:

$$\Delta R_{x}^{BE}=\sum_{i=0}^{n}\left(A_{i}^{bound}-A_{i}^{free}\right)$$ (3)

Where $A_{i}^{bound}$ and $A_{i}^{free}$ are the energy of *ith* atom from *x* residue in bound and free forms, respectively, and *n* is the total number of atoms in the residue.

The *g_mmpbsa* module does not include the calculation of entropic terms and therefore it gives the relative binding energy. Since we have used the same compound (Imatinib) interacting with proteins of similar structures and showing the same binding mode, the entropy contribution was neglected [62].

### Data simulation analysis

#### Electrostatic potential surface

The electrostatic potential surface was computed with the APBS software running through the PDB2PQR webserver (http://nbcr-222.ucsd.edu/pdb2pqr/). As the input data, the equilibrated conformations of the Imatinib-target complexes prior to MD simulations, were used.

#### Salt bridges

The salt bridges occurrences were calculated for the merged trajectories containing 9 000 frames using the *Timeline plugin* available with VMD. The residues-pair selection was made within a cutoff distance of 10 Å from Imatinib. Plots were generated using R software [63], discarding interactions occurring less than 10% of the simulation time.

### Inhibition of KIT phosphorylation

#### Cell transfection

COS-7 cells were transfected with 1 μg of KIT^WT^, KIT^D816V^, KIT^V560G^ or KIT^S628N^ expression vectors with FuGENE 6 (Promega). Twenty four hours following transfection, cells were split in four 60-mm plates and serum-starved overnight in Dulbecco’s modified Eagle’s medium with 0.5% FBS. Cells were then treated for 90 minutes with Imatinib at concentrations ranging from concentration 0 to 5μM. Cells transfected with hKIT WT were stimulated for 5 min with 250 ng/ml murine SCF at 37°C.

#### Immunoblotting

Cells were washed in ice-cold phosphate-buffered saline (PBS), pelleted, and lysed in HNTG buffer (50mM HEPES [pH 7], 50mM NaF, 1mM EGTA, 150mM NaCl, 1% Triton X-100, 10% glycerol, 1.5 mM MgCl2) containing the protease inhibitor mix Complete EDTA-free (Roche Applied Science) and 100 μM Na_3_VO_4_. Proteins were separated on SDS-polyacrylamide gels and transferred to Immobilon-P membrane (Millipore). Membranes were saturated with 5% bovine serum albumin (Euromedex) and probed with phospho-c-Kit (Tyr719) (Cell Signaling Technology) and c-Kit (D13A2) antibodies (Cell Signaling Technology). Signals were revealed using horseradish peroxidase-conjugated secondary antibodies and SuperSignal West Pico Chemiluminescent Substrate (ThermoScientific).

## Results and Discussions

### Imatinib docking into the targets

For the study of stability of the Imatinib•target complexes and the comparison of their free binding energy, we used the representative conformations of the inactive form of native (or wild-type, WT) and mutated kinase domain of two proteins, CSF-1R (CSF-1R^WT^ and CSF-1R^D802V^) and KIT (KIT^WT^, KIT^D816V^, KIT^V560G^ and KIT^S628N^), selected from our previous MD simulations [40–41, 43–44]. Our earlier attempts to dock Imatinib into the inactive form of the kinase domain of KIT and CSF-1R have failed [64], probably due to the buried configuration of the JMR. To clarify the reason of this failed assay, we compared the crystallographic structures of KIT and CSF-1R bound and non-bound to Imatinib– 1T46 [25], 4R7I [65], 2OGV [66] and 1T45 [25], respectively. We observed that the JMR buried configuration and the side-chain orientation of residues W550/557 and F798/811 (CSF-1R/KIT) in both receptors, impairs the binding of Imatinib into the ATP-binding site (Fig 1E). Repositioning of the side chain of F811/797 in KIT/CSF-1R bound to Imatinib, evidenced by crystallography (1T46/4R71), confirms its inhibitor-induced (or inhibitor-selected) relocation. The Imatinib binding effect on the JMR residue W550/557 (CSF-1R/KIT) inserted in the binding pocket remains obscure, because the N-extremity of the JMR is not resolved in the crystallographic structures 1T46 and 4R7I, suggesting that this portion might be very flexible and dislocated from the kinase domain. To avoid steric hindrance, we removed the JMR from the selected MD conformations, producing suitable targets for the docking procedure. In the mutant KIT^V560G^, to conserve the point mutation, the JMR was truncated at position 558, yielding a target, longer by 30 residues (V558-R588) than the other targets. Expecting a tiny impact of this JMR partial fragment on Imatinib binding, we have accepted such exception to compare the Imatinib binding to all studied targets.

**Fig 2:**
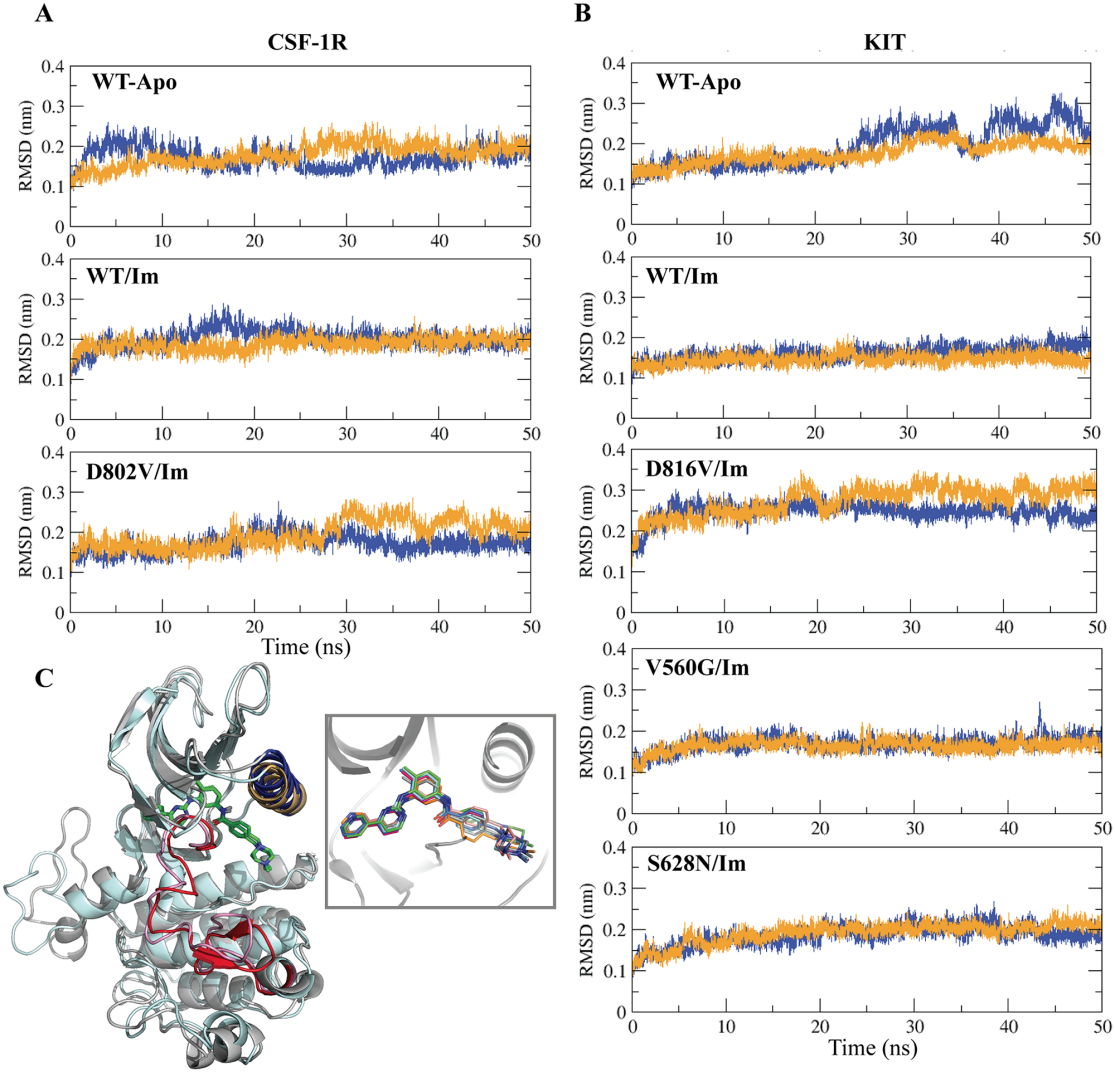
MD simulations of the RTK-Imatinib complexes. (**A-B**) The Root Mean Square Deviation (RMSD) values of the native non-bonded receptors (CSF-1R^WT-APO^ and KIT^WT-APO^) and the complexes formed by Imatinib and the native receptors (CSF-1R^WT^ and KIT^WT^) and their mutants (CSF-1R^D802V^, KIT^D816V^, KIT^V560G^ and KIT^S628N^) were calculated for the backbone atoms from trajectories 1 and 2 (orange and blue respectively) of MD simulations of all studied systems. (**C**) Simulated complexes were obtained by the docking of Imatinib (in green) to the active site of each receptor. The JMR was removed prior the docking procedure. The superimposed receptors CSF-1R/KIT are shown as grey/cyan cartoons. The key structural fragments of receptors, the αC-helix and the activation loop (A-loop) are highlighted in cyan/brown (CSF-1R/KIT) and in red/rose (CSF-1R/KIT) color respectively. **Insert** in **C**: The best docking poses of Imatinib bound to CSF-1R^WT^(blue), CSF-1R^D802V^(orange), KIT^WT^(cyan), KIT^V560G^(green), KIT^S628N^(pink) and KIT^D816V^(magenta) superposed with the crystallographic structure 1T46 (grey). The protein backbone and Imatinib are represented in cartoon and sticks, respectively.

The optimised structure of the protonated Imatinib [26], generated with *Ligprep*, was successfully docked into each prepared target (see Methods). The best docking poses of Imatinib show a striking similarity in all studied proteins (Fig 2D, insert) and all are very close to that observed in the crystallographic structure of Imatinib•KIT (1T46) and Imatinib•CSF-1R (4R7I) complexes, in which the position of Imatinib and its conformation is virtually identical (Fig A in S1 File). Taking 1T46 as a reference, the docking poses, showing Root Mean Square Deviation (RMSD) values less than 1 Å and reproducing five H-bonds described in the crystallographic structure, were preselected. The best poses, ranked by RMSDs and GLIDE scores, were retained for further analysis. The GLIDE scores of the best poses, ranging from -9.2 to -6.3 kcal/mol over the generated complexes, showed a common tendency to be diminished (higher affinity) in the native proteins and KIT^V560G^, and worse in the resistant mutants (Table 1). Although GLIDE docking scores show only qualitative correlation with the experimentally determined Imatinib affinity, their values help the choice of ‘Imatinib•target’ complexes further studied by MD simulations. The IFD scores, distributed in a narrower range, from -12.78 to -10.96 kcal/mol, do not significantly distinguish the binding affinity of Imatinib to the different targets.

**Table 1:** Imatinib docking into the native targets (CSF-1R^WT^ and KIT^WT^) and their mutants (CSF-1R^D802V^, KIT^V560G^, KIT^S628N^ and KIT^D816V^). The two energy values, the GLIDE and Induced Fit Docking (IFD) scores, were produced with MAESTRO (Schrödinger release, 2014). The RMSD values were calculated for the predicted docking poses taking crystallographic structure 1T46 as a reference. The IC_50_ values (μM) are taken from the literature.

Target/Parameter	Score Glide (kcal/mol)	Score IFD (kcal/mol)	RMSD (Å)	IC_50_^Reference^ (μM)
CSF-1R^WT^	-7.9	-10.9	0.5	0.3 [42]
CSF-1R^D802V^	-6.3	-12.4	0.5	>4 [42]
KIT^WT^	-7.8	-12.2	0.5	0.1–1 [18,19]
KIT^V560G^	-9.2	-12.1	0.6	0.025 [18]
KIT^S628N^	-8.6	-12.3	1.0	not determined
KIT^D816V^	-7.0	-12.1	0.4	>10 [19]

### Molecular dynamic simulations of CSF-1R and KIT complexes

To get further insight into the molecular mechanisms of the resistance to Imatinib, it is of primary importance first to investigate the structural and dynamical properties of the Imatinib-bound targets and second, to define the energy-related parameters driving the molecular complex formation and stability. For these purposes, we performed MD simulations on Imatinib-bound receptors, KIT and CSF-1R, in the native and mutated forms.

#### Stability of the target•Imatinib complexes

The RMSDs calculated on the backbone atoms from the initial structures of CSF-1R and KIT in their free and bound states are relatively stable over MD simulations, except the very flexible residues located at the C-terminal loop of both receptors, which are significantly contributing to the RMSD values increase. These residues (7/6 residues from the C-terminal of CSF-1R/KIT) were excluded from the RMSD analysis. Overall, the RMSD values in almost all simulated complexes vary around 0.15–0.20 nm (Fig 2 and Fig B in S1 File), while one of the two MD simulations replica of each receptor possessing the equivalent mutation, D802V (CSF-1R) or D816V (KIT), shows slightly increased RMSD values (0.25 and 0.35nm, respectively).

**Fig 3:**
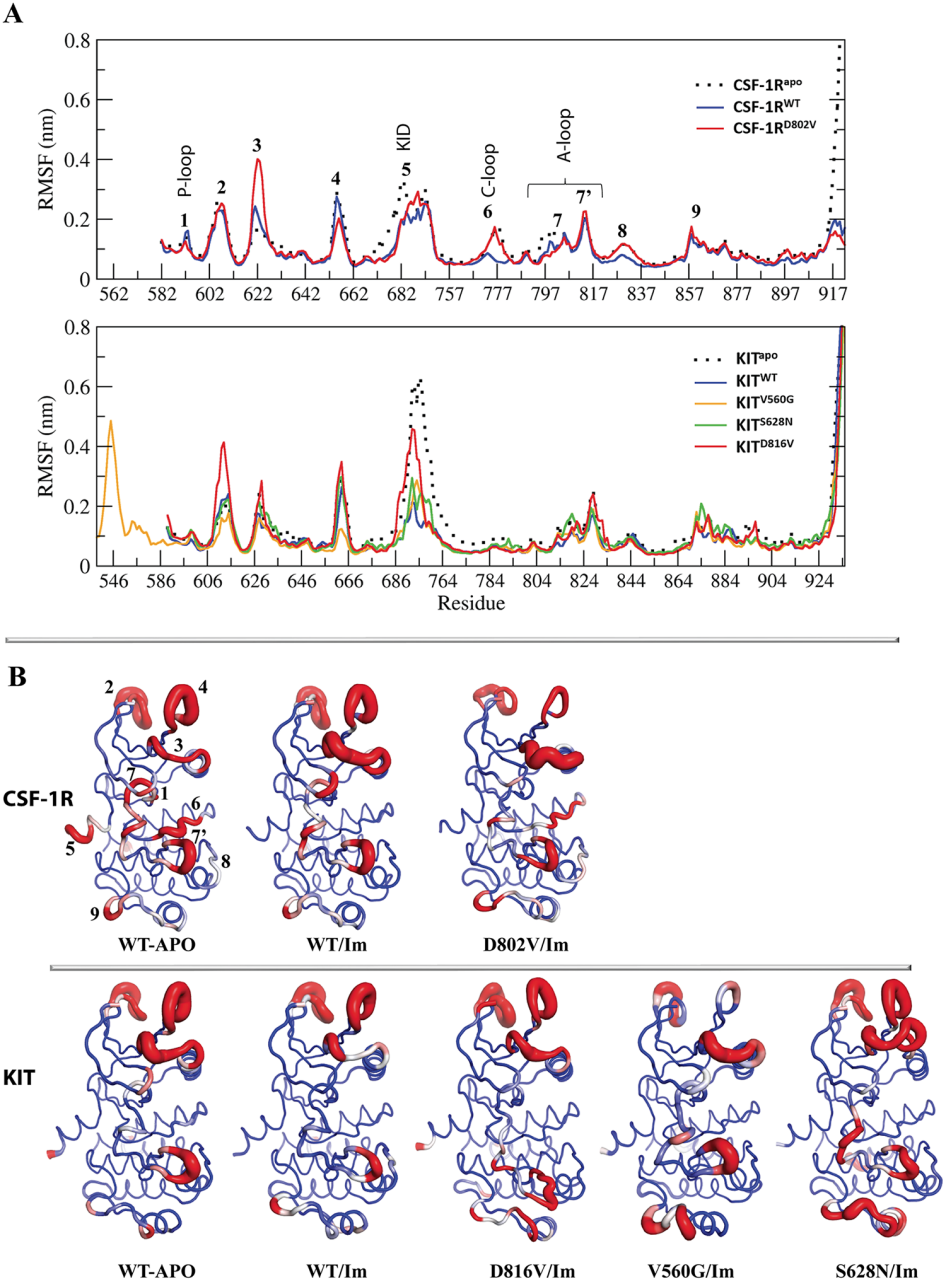
MD simulations of the RTK-Imatinib complexes. (**A**) The Root Mean Square Fluctuations (RMSFs) computed on the backbone atoms averaged over the total production simulation time of CSF-1R^WT^ and CSF-1R^D802V^ (blue and red, respectively) (*top panel*) and of KIT^WT^, KIT^D816V^, KIT^V560G^ and KIT^S628N^ (blue, red, yellow and green, respectively) complexes (*bottom panel*) were compared to those in the non-bound receptors (dotted black lines). (**B**) The average conformations of CSF-1R (*top panel*) and KIT (*bottom panel*) are presented as tubes. The tube size is proportional to the residue atomic fluctuations computed on the backbone atoms. The highly fluctuating regions are colored in red and numerated for CSF-1R in (**A**) and (**B**).

To identify the residues responsible for this increased structural instability, we computed the Root Mean Square Fluctuations (RMSFs) of all amino acids in each protein. The MD RMSFs, usually coupled with the 3D interpretation of the temperature factors (beta-values) from crystallographic data, show that the highest atomic fluctuations are principally observed, as was expected, in some particular loop regions, as we established previously in the non-bonded targets [41,43,44]. Comparing the native non-bound targets, CSF-1R and KIT, we note some differences in the atomic fluctuations of residues from the molecular fragments involved in the activation process—the catalytic (C-) loop, the activation (A-) loop and the loop preceding αC-helix, annotated on Fig 3 as **6**, **7** and **3**, respectively. When analysing the Imatinib-bound complexes, we remarked that binding of Imatinib influences differently the atomic fluctuations of the native and mutated targets.

In both Imatinib•CSF-1R complexes, formed by the native and mutated receptors, we observed an alteration of atomic fluctuations respectively to those in the non-bound target—higher for residues of the loop preceding the αC-helix (**3** in Fig 3), and diminished for residues of the 3^10^-helix of A-loop (**7** in Fig 3). The RMSFs of the N-lobe loops residues (**2** and **4** in Fig 3), showing similar values in the non-bound target (CSF-1R^WT/APO^) and in Imatinib•CSF-1R^WT^ complex, are significantly diminished in the complex formed by the mutated target CSF-1R^D802V^. The P-loop residues (annotated as **1** in Fig 3) show diminished RMSFs in complexes compared to non-bound receptor.

In KIT complexes, the RMSFs pattern generally corresponds to that in CSF-1R’s (Fig 3). Nevertheless, some features, characterizing the atomic fluctuations in KIT, mutated or not, bound or not to Imatinib, were different from those in CSF-1R’s. In particular, in the non-bound native KIT, the RMSFs of residues from the loop (**3**) preceding the C-helix are increased respective to those in CSF-1R, whereas they are significantly diminished in the A-loop 3^10^-helix (**7**) and in the catalytic (C-) loop (**6**). When KIT is bonded to Imatinib, the RMSF values in the N-lobe’s loop preceding the C-helix are diminished in the native target—the effect opposite to those observed in CSF-1R–, while in complexes formed by the mutants theirs values are conserved. For the Imatinib-bound KIT^V560G^ and KIT^S628N^ mutants, specifically, the increasing of RMSFs is observed for residues from the A-loop (annotated as **7’** in Fig 3) and the C-lobe loop preceding G-helix (**9** in Fig 3). One of the differences between the two receptors, KIT and CSF-1R, is the atomic fluctuations of the catalytic (C-) loop residues. These values are diminished in both CSF-1R complexes compared to the non-bound target—significantly in Imatinib•CSF-1R^WT^ and moderately in Imatinib•CSF-1R^D802V^. Apparently, the increase of the C-loop atomic fluctuations in Imatinib•CSF-1R^D802V^ complex is directly related with the alteration in the H-bonds pattern involving residues of the C- and A-loops (Table 2). In all KIT targets, wild-type and mutated, bound or not, the RMSFs of the catalytic (C-) loop are similarly very small.

**Table 2:** H-bonds between the A- and C-loops residues in CSF-1R and KIT. Occurrences (in %) of the H-bonds are averaged over the two MD replicas.

Contact/Target	CSF-1R^WT^	Im•CSF-1R^WT^	Im•CSF-1R^D802V^
D778⋯N783	92	100	70
D778⋯H776	69	60	67
D778⋯R801	93	78	1
D778…Y809	18	39	78
D778⋯R782	2	7	94
Contact/Target	KIT^WT^	Im•KIT^WT^	Im•KIT^D816V^
D792⋯N797	50	51	89
D792⋯H790	23	34	43
D792⋯R815	100	100	74
D792⋯Y823	47	45	45

#### The H-bonds pattern stabilizing the Imatinib binding in the complexes

**Fig 4:**
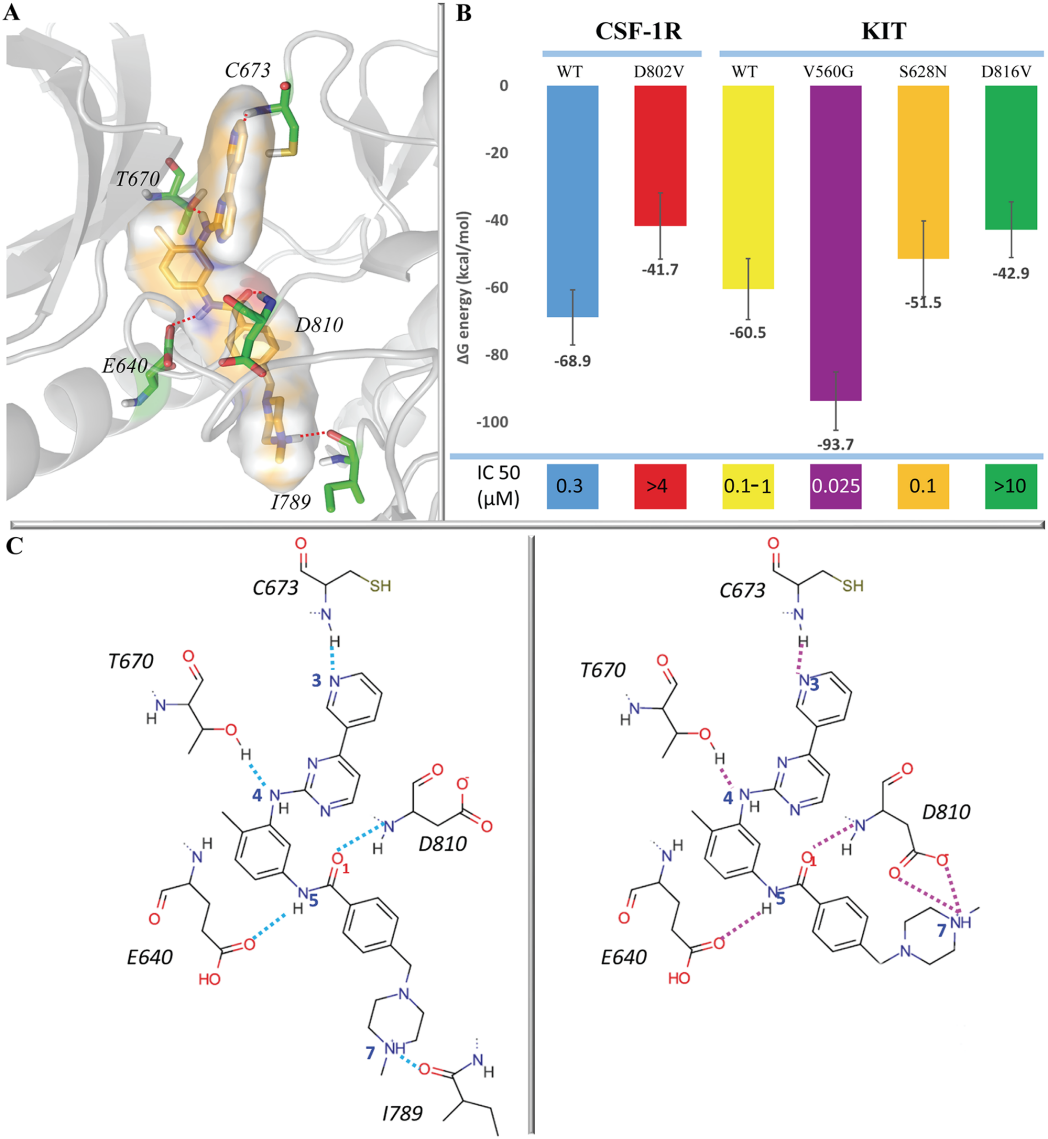
Imatinib binding to CSF-1R and KIT. **(A)** 3D structure of Imatinib•target complex illustrated by using KIT^WT^ bound with Imatinib. Imatinib and interacting residues of receptor are showed by sticks. (**B**) Graphical representation of the free binding energy (ΔG) of Imatinib•target complexes. The computed total ΔG energy value (this work) and the experimentally measured affinity (the literature data and the present data) are shown for each complex. (**C**) The H-bonds (dashed lines) pattern stabilizing Imatinib in complexes formed by the native KIT and its mutants KIT^D816V^ and KIT^V560G^ (left), and in mutant KIT^S628N^ (right).

The five H-bonds observed in the crystallographic structures (1T46 and 4R7I for KIT and CSF-1R, respectively) and described as the principal non-covalent contacts stabilising the binding of Imatinib to the native targets (KIT^WT^ and CSF-1R^WT^), are well preserved along MD trajectories (Fig 4A and 4C, scheme on the left). These H-bonds are formed by functionally significant amino acids in KIT^WT^/CSF-1R^WT^–the gatekeeper residue T670/663, the highly conserved residue E640/633, the DFG residue D810/796, the hinge residue C673/666 and the C-loop residue I789/775. Over MD simulations of Imatinib•KIT^WT^/CSF-1R^WT^ complexes, these H-bonds are characterized by high, moderate or low occurrence values– 98% (C673/666), 47/38% (T670/663), 42/37% (D810/796), 12/34% (E640/633) and 96/98% (I789/775), respectively (Table 3, Scheme A in S1 File).

**Table 3:** Imatinib binding to the targets. Occurrences of the H-bonds between Imatinib and the targets residues were averaged over each of the two MD replicas. Imatinib atoms participating in the interactions with the targets are represented in Scheme A in S1 File. The atom pairs for donor-acceptor interactions are depicted in the Table.

Target/Atoms	N3-(N)C666	O1-(N)D796	N7-(Oδ)D796	N7-(O)I775	N5-(Oε)E633	N4-(Oγ)T663
CSF-1R^WT^	98	37	0	96	34	38
CSF-1R^D802V^	99	26	0	18	34	51
	N3-(N)C673	O1-(N)D810	N7-(Oδ)D810	N7-(O)I789	N5-(Oε)E640	N4-(Oγ)T670
KIT^WT^	98	42	0	98	12	47
KIT^V560G^	98	73	0	95	46	34
KIT^S628N^	93	68	56	33	34	27
KIT^D816V^	98	60	0	99	27	51

This analysis shows that in KIT and CSF-1R complexes formed by the native targets, the H-bonds of Imatinib with the hinge residue C673/666 and with the hydrophobic residue I789/775 are constantly and similarly maintained during the MD simulations with an occurrence close to 100%. The other H-bonds, characterised by relatively lower occurrences, show some difference between the two targets. In particular, the H-bond of Imatinib with D810 in KIT shows higher occurrence (42%) than compared to E640 (12%), while the corresponding H-bonds in CSF-1R have similar occurrence. The H-bonds involving the residue T670/663 have close occurrences in both native targets.

As it was evidenced by the analysis of different X-ray structures, all these H-bonds stabilizing Imatinib in the binding site are observed in complexes formed by KIT, CSF-1R and other tyrosine kinases, while the author’s interpretation of the observed contacts may differ. For instance, in the crystal structure of KIT complexed with Imatinib (1T46) [25], the Imatinib interactions with I789 through the N-methylpiperazine fragment was described as non-specific. In the recently solved structure of Imatinib in complex with CSF-1R (4R7I) [3], this contact is considered as alternative to that with H776. In other structures of kinases complexed with Imatinib, such as Abl (2HYY) [67], c-Src (2OIQ) [22] and DDR1 (4BKJ) [68], the N-methylpiperazine fragment of Imatinib makes direct contacts with a hydrophobic residue (isoleucine or valine) and with histidine. Such interpretation of the N-methylpiperazine moiety’s stabilising contacts is probably related to considering the Imatinib as a neutral molecule over the structure refinement in the X-ray analysis. Therefore, our interpretation of Imatinib contact with I789 as a strong and stable H-bond interaction should be considered for the improving or the development of kinase inhibitors.

In CSF-1R^WT^ and CSF-1R mutant, the H-bond interactions stabilizing Imatinib in its binding site are overall conserved; however, their occurrence values vary significantly among the different mutants (Table 3). In particular, an essential decrease of the H-bond occurrence is detected for the interaction of Imatinib with I775 in CSF-1R^D802V^ (18% against 96% for CSF-1R^WT^. In KIT ^D816V^ the occurrences for the same H-bonds were surprisingly maintained. I775/789 (CSF-1R/KIT) is placed in the C-loop, and the binding of this residue with Imatinib correlates directly with the C-loop RMSFs observed in the related proteins.

As expected, given its higher sensitivity to Imatinib compared to KIT^WT^, KIT^V560G^ binds Imatinib through H-bonds characterised by higher occurrences than in KIT^WT^, particularly for interactions with D810 (73% vs 42%) and D640 (46% vs 12%). Some change in occurrences of the H-bond interactions was also evidenced in the Imatinib•KIT^S628N^ complex, formed by a mutant recently found in a metastatic melanoma and described as still sensitive to Imatinib [43]. In this complex, mutation affects mainly the inhibitor interactions with residues I789 and D810, which are reduced for I789 (33% vs 98%) and increased for D810 (68% vs 42%). Furthermore, Imatinib interacts with residue D810 by H-bond with the δ-oxygen atom in KIT^S628N^, a contact that was not observed in complexes formed by the other targets (Table 3, Scheme A in S1 File). Visual inspection of the Imatinib•KIT^S628N^ MD conformations showed that this H-bond occurs due to a flip of the N-methylpiperazin fragment of Imatinib and a change on the side chain orientation of D810, favouring the interaction between the inhibitor and the D810 δ-oxygen atom (Fig 4, scheme on the right). Such conformational adaptation of both interacting partners, target and inhibitor, induces a significant diminishing or entire loss of the inhibitor interaction with I789 (33% KIT^S628N^ vs 98% in KIT^WT^) but stabilizes the bifurcate H-bond with D810 side chain. For each complex, the H-bonds patterns stabilizing the Imatinib-target binding and the H-bonds occurrences were similar when either the individual or concatenated MD trajectories were analyzed (Table 3, Table A in S1 File).

### The free binding energy in Imatinib•target complexes and its relation with targets sensitivity to Imatinib

The conformational change in the ATP-binding site is a footprint of the induced-fit mechanism according of which the protein adapts its active site conformation to accommodate the ATP or inhibitor. Previously, based on normal modes analysis, it was concluded that KIT mutants (including D816V) had a decrease in flexibility of the majority of residues that participated in the binding of Imatinib [39]. In our MD simulations, the H-bond pattern found for KIT^D816V^ indicates an increased rigidity of the ATP-binding site. Due to the reduced flexibility, residues forming the ATP-binding site in KIT^D816V^, would be less able to adapt their conformation over the recognition process, possibly reducing the affinity for the inhibitor. In contrast, the conformational adaptation of mutated target (in Imatinib•KIT^V560G^) or together with the inhibitor (in Imatinib•KIT^S628N^) may be sufficient to reach a good affinity between two partners.

#### Experimental evaluation of KIT sensitivity to Imatinib

**Fig 5:**
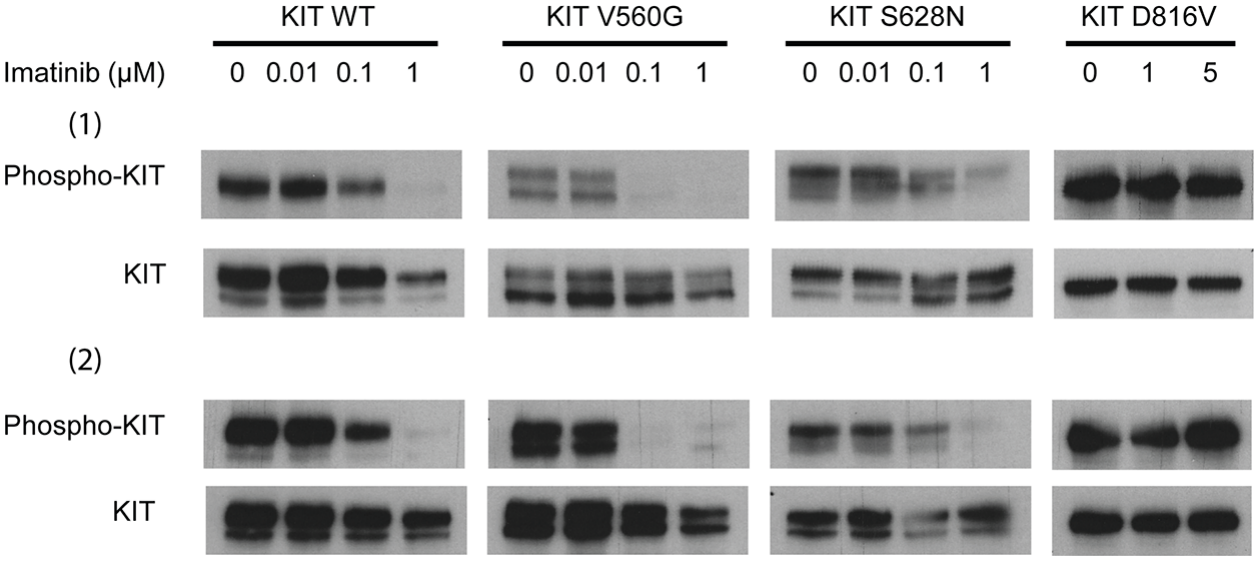
Inhibition of KIT phosphorylation by Imatinib. Cells expressing KIT^WT^, KIT^D816V^, KIT^V560G^ or KIT^S628N^ were treated with the indicated concentrations of Imatinib for 90 minutes. Protein lysates were analyzed by western-blotting to reveal KIT protein expression (KIT) and KIT phosphorylation (Phospho-KIT) as readout of Imatinib inhibition. Two independent experiments were performed.

To clarify the role of the S628N substitution in KIT, we determined experimentally the relative sensitivity of the various KIT (KIT^WT^, KIT^D816V^ KIT^V560G^ and KIT^S628N^) receptors. COS7 cells were transfected with the corresponding expression vectors, and were then treated with various concentrations of Imatinib ranging from 0.01 to 5 μM. The inhibition of KIT phosphorylation was then evaluated by western-blotting (Fig 5). As previously described, KIT^D816V^ was not affected even at 5 μM, while KIT^WT^ was partially inhibited at 0.1 and fully inhibited at 1 μM. As expected also, KIT^V560G^ was inhibited at 0.1μM. Finally, KIT^S628N^ had an inhibition comparable to KIT^WT^. Consequently, the experimental data indicates the following order of KIT sensitivity to Imatinib: KIT^V560G^> KIT^WT^ = KIT^S628N^>> KIT^D816V^.

#### The binding free energy of Imatinib•target complexes

Since the H-bonds pattern does not explain exhaustively a difference of the Imatinib binding to the sensitive and resistant targets, we further analysed the binding free energy, (ΔΔG), [69] of Imatinib to each target using the MM/PBSA approach [59]. The binding free energy values are considerably different in the studied complexes (Fig 4B). The lowest (-94 kcal/mol) and the biggest (-42/-43 kcal/mol) values are found in complexes formed by the most sensitive (KIT^V560G^) and the most resistant (CSF-1R^D802V^ and KIT^D816V^) targets to Imatinib, respectively. Based on these binding free energy values, the *in silico* estimated sensitivity of the studied targets to Imatinib may be described by the following order—KIT^V560G^ > CSF-1R^WT^/KIT^WT^ ≥ KIT^S628N^ ≥ CSF-1R^D802V^/KIT^D816V^ and CSF-1R^WT^/KIT^WT^ > CSF-1R^D802V^/KIT^D816V^, which is qualitatively very consistent with our *in vitro* data.

In general, the experimentally measured sensitivity of the different KIT receptors to Imatinib shows the same tendency as predicted by the free energy of binding calculations. Only the KIT^S628N^ energy profile could not be significantly distinguished *in silico* from those of KIT^WT^ and KIT^D816V^. Such divergence between the theoretical prediction and the experimentally measured values may be attributed to the limitation of the MM/PBSA method, in comparison to more accurate approaches such as alchemical free energy perturbation molecular dynamics (FEP/MD) [70].

However, high absolute quantitative accuracy was not in the scope of the present study that focuses on the discrimination of Imatinib binding by the native and mutated targets (RTKs). To our knowledge, this demonstration of a difference in the energy of binding of Imatinib to the native and resistant forms of these two kinase targets, evidenced computationally, is new. The binding energy alteration in Imatinib-targets complexes together with the earlier described mutation-induced perturbation of the Imatinib-specific inactive conformation as an important step for transition toward the constitutively active form [41,44], fully explain the resistant profile of the studied CSF-1R^D802V^/KIT^D816V^ mutants.

Since Imatinib is held in the kinase pocket via an extended network of hydrogen bonding and charge-charge interactions, the decomposition of the binding free energy on the different terms contributing to the binding affinity of Imatinib helps to identify the primary forces driving such binding and to analyze their variations among different complexes. The electrostatic energy (ΔG_elect_) shows more variation (from -106 kcal/mol in KIT^V560G^ to -54 kcal/mol in CSF-1R^D802V^) than the others terms and appears to be the principal force contributing to the difference in the final binding energy values (ΔG_bind_) in the studied complexes (Table 4, Table B in S1 File). The correlation coefficient between ΔG_elect_ and ΔG_bind_ is 0.89. The solvation energy (ΔG_sol_) and the van der Waals dispersion energy (ΔG_vdw_) show only minor variations among the studied complexes (ΔΔG_sol_ and ΔΔG_vdw_ is less than 10 and 3 kcal/mol, respectively). The significant role of the electrostatic component in the Imatinib binding affinity to KIT was reported earlier [27]. Recently, the analysis of the reported literature binding energies of Imatinib to tyrosine kinases, obtained by molecular mechanics (MM) and those found by quantum mechanics (QM), suggested that solvation energies are a major component of the overall binding energy [71].

**Table 4:** The free energy of binding in the Imatinib-targets complexes. The free binding energy (ΔG_bind_) and contributions of electrostatic (ΔG_elec_t), van der Waals (ΔG_vdw_) and solvation (ΔG solv) energies were calculated using MM-PBSA. All energies are in kcal/mol.

RTK	ΔG_vdw_	ΔG_elect_	ΔG_sol_	ΔG_bind_
CSF-1R^WT^	-66	-76	73	-69
CSF-1R^D802V^	-66	-54	78	-42
KIT^WT^	-67	-75	81	-61
KIT^D816V^	-66	-56	80	-43
KIT^V560G^	-64	-106	76	-94
KIT^S628N^	-67	-67	83	-51

In our quite simply analysis, we have shown that KIT ^V560G^ has the most favorable protein-Imatinib electrostatic interactions, greater than the native targets, KIT and CSF-1R, suggesting that mutation V560G promotes the largest electrostatic stabilization of the Imatinib binding. On the contrary, mutation D802V in CSF-1R and D816V in KIT, diminishes the electrostatic interactions with Imatinib.

#### The per residue energy contributions to the binding energy

To identify the key molecular determinants responsible for Imatinib binding to the targets, we calculated per residue energy contribution of all protein residues to the overall binding energy. As was expected, the free energy binding value is mostly influenced by the charged residues,–either favorably (impact of the negatively charged amino acids, asparagine and glutamic acid)–or unfavorably (impact of the positively charged residues, arginine and lysine) demonstrating nearly similar contributions from the corresponding residues in CSF-1R and KIT (Tables C and D in S1 File). The substitution of an aspartate for a valine (D→V) in CSF-1R^D802V^ and KIT^D816V^ reduces significantly the residue energy contribution—from -8 kcal/mol (CSF-1R^WT^/KIT^WT^) to 0 kcal/mol (CSF-1R^D802V^/KIT^D816V^). In KIT^V560G^, the residue V560G, distant by ~8 Å from the Imatinib, does not contribute considerably (-0.3 kcal/mol) to the binding energy. Similarly, in KIT^S628N^, the native or mutated residue affects lowly the binding energy. This estimation also evidenced that the equivalent Imatinib-binding site residues (in CSF-1R and KIT), making direct contact with the inhibitor, contribute almost similarly to the binding energy and show only small differences between the native and mutated targets.

#### The JMR role in the Imatinib binding

Since the ΔG_bind_ energy associated with the binding of Imatinib to KIT^V560G^ is low compared to the native protein and to the other studied targets, we evaluated the role of the partial JMR fragment, considered for the calculations in this mutant. The newly generated MD trajectories of the Imatinib•KIT complexes (where KIT = KIT^WT^, KIT^S628N^ and KIT^D816V^, having the same portion of the JMR (partially cleaved) as the mutant KIT^V560G^), were used for the ΔG_bind_ energy calculation.

Comparing the data (MM-PBSA) obtained for Imatinib•KIT complexes formed by (I) the targets having the partial JMR (Fig C in S1 File) and (II) the targets with the entirely cleaved JMR (Fig 4B), we observed that (i) the tendency of the computed free energy of binding in the two types studied complexes, formed by either the targets with totally cleaved JMR or having partially cleaved JMR, is maintained; (ii) the free binding energy values are systematically lower in all complexes of type (I) in respect to those of type (II); (iii) the difference in free energy binding between the imatinib-bound complexes formed by KIT^D816V^ and KIT^WT^ consists of 20–30% in the two types of complexes (I) and (II); (iv) the values of ΔG_bind_ in complexes formed by KIT^S628N^ and KIT^WT^ targets, having the partial JMR, are close. All these observations indicate that the length of receptor, of type (I) or (II), influences the free energy values but has only a tiny impact on the qualitative results distinguishing the energy binding to the different targets. The last observation, (iv), corresponds well to the experimentally measured data that indicate the similar sensitivity of these proteins to Imatinib.

#### Contributions of residues positioned out of the ATP-binding site to the binding energy

**Fig 6:**
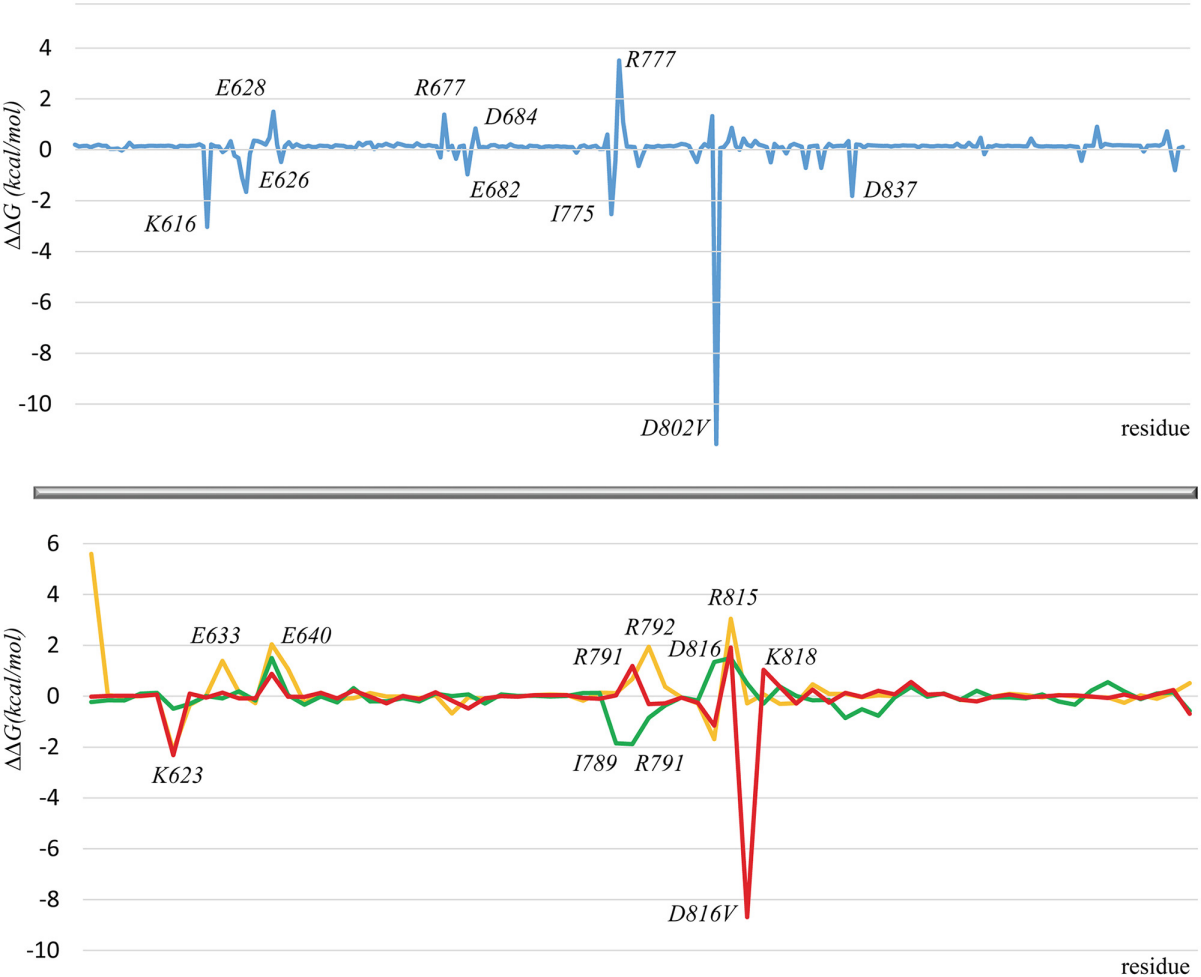
Binding energy of the Imatinib•target complexes. Difference in the per residues contribution to the binding energy (ΔΔG) between the native and mutated targets–(**top**) CSF-1R^WT^ and CSF-1R^D802V^(blue), and (**bottom**) KIT^WT^, KIT^V560G^ (orange), KIT^S628N^ (green) and KIT^D816V^ (red). The labeled residues are characterized by ΔΔG values more than 1 or less than -1 kcal/mol.

Considering the inhibitor-target recognition as a cooperative process, we showed that the protein residues positioned at the vicinity of the ATP-binding site might affect the global binding energy. We have subtracted the energy contributions of the residues from mutant with those of the native protein, and the obtained increments (ΔΔ*G*), ‘positive’ or ‘negative’, are illustrated in Fig 6.

In CSF-1R, except the most important impact of D802, several residues participate to either increase the unfavorable contribution (K616) or reduce the favorable contribution (E626, I775 and D837) to the binding energy. With exception of I775, all these residues are not bound directly to Imatinib (Fig 4). As a ‘positive’ effect on the Imatinib-CSF-1R recognition, we noted only a diminishing of the unfavorable contribution (R777) and a small increasing of the favorable contribution (E628) in the mutant respectively to the native protein. The difference in the residual contributions (ΔΔG) in KIT^D816V^, in general, is close to those in CSF-1R^D802V^. In particular, the residues contributing negatively to the Imatinib binding, besides of the important impact of the D816V substitution, are D810, making H-bond with Imatinib, and K623, not interacting directly with Imatinib.

Such repartition of the favorable and non-favorable contributions resulted in a global diminishing of Imatinib affinity to CSF-1R^D802V^ and KIT^D816V^ mutants, explaining its resistance to the drug. Curiously, all H-bonds stabilizing Imatinib binding by KIT are preserved in KIT^D816V^ mutant. Moreover, D810 forms more prevalent H-bonds (60%) in Imatinib• KIT^D816V^ complex compared to Imatinib• KIT^WT^. It is not excluded that in KIT, together with electrostatic interactions, the dispersion contribution is also important in the Imatinib binding. According to the literature, in KIT^WT^, the disperse interaction energy was estimated to be -7.1 kcal/mol, while the electrostatic contribution is 4.7 kcal/mol [27]. Moreover, the water molecules localized in the binding pockets may contribute significantly to the Imatinib affinity, as was reported in this study. Despite the H-bond pattern conservation, the overall energy in the Imatinib complex formed by the KIT mutant is considerably changed as we demonstrated using MM/PBSA method. A more detailed computation based on alchemical FEP/MD simulations could be useful to investigate further the molecular determinants of the KIT^D816V^ resistance.

Comparing KIT^WT^ and KIT^V560G^, we noted that the ‘positive’ increment in the binding energy of the mutant is furnished by residues E633, E640, D792, and R815 that contribute significantly to the Imatinib affinity either by impact of the negatively charged residues E633, E640 and D792 or by diminishing of the unfavorable interaction by R815. Such increase of the favorable interactions could be a main factor contributing to the higher affinity of Imatinib to KIT^V560G^ compare to the native target (Fig 4B). In this protein, a partial JMR may affect the global free binding energy, contributing favorably to the ΔΔ*G* value in the mutant (Table C and Table D in S1 File). The energy contribution of the non-ATP binding site residues increases the global energy binding of all KIT targets, decreasing the ΔΔ*G* value, in particular for the sensitive mutants KIT^WT^ and KIT^S628N^.

In Imatinib•KIT^S628N^ complex, two residues, I789 and R791, located in the C-loop, supply the most significant positive decrement to the binding energy, while residue D810 contributes to the ‘negative’ increment (Fig 6). The impact of these two opposite effects is compensated completely. Moreover, we observed that during the MD simulations of this complex, the methyl-piperazinyl moiety of Imatinib abolished its interaction with I789 and established a bifurcated H-bonding with D810 (Fig 4C). As already discussed, such alternation of the H-bond pattern either is induced by a flipping of the methyl-piperazinyl fragment inside the ATP-binding site or promotes such flipping, suggesting the induced fit of imatinib by the ATP-binding site adjustment.

#### Formation of the salt-bridges in Imatinib•target complexes

Since we observed that the charged residues have the highest impact on the binding energy contributions in all studied complexes, we suggested a possible alteration of the salt-bridges in the mutants, especially in the vicinity of the Imatinib-binding site. The salt-bridges, a peculiar type of non-covalent interaction, combines two main components—hydrogen bond and electrostatic interaction—between two ionized sites.

**Fig 7:**
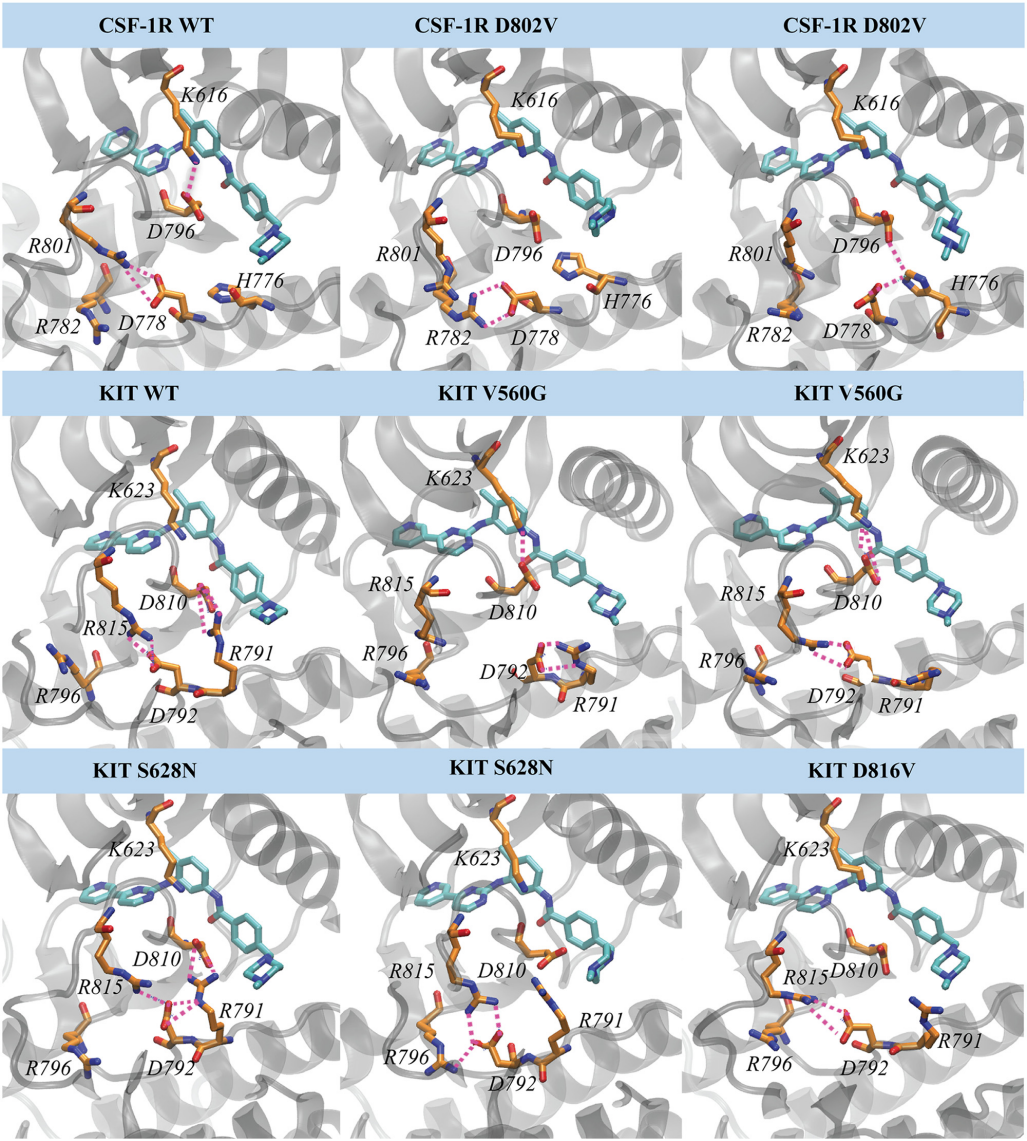
Salt-bridges at proximity of the binding pocket calculated over the MD simulations of the Imatinib•target complexes. Representative conformations of the proteins are shown as cartoon, Imatinib (in blue) and residues forming salt-bridges (in orange) are drawn in stick. Non-covalent interactions are shown by dashed lines.

Comparing the salt-bridges formed in the binding site of CSF-1R^WT^ and CSF-1R^D802V^, we observe that the salt-bridges D778•••R801 and D796•••K616, established in the native protein, were completely disrupted in the mutant (Fig 7). As was identified through the MM-PBSA computing, the residues establishing salt-bridges in CSF-1R are important contributors to the total free binding energy with Imatinib, especially K616 (Table A in S1 File). All these residues are located at proximity of the Imatinib binding pockets, in particular, D778 is in the C-loop, D796 and R801 in the A-loop, and K616 in the β3 turn. The perturbed orientation of the side chains of R801 and D796 in CSF-1R^D802V^ mutant, correlated to the changed atomic fluctuations (RMSFs), promotes their participation in an alternative salt-bridges pattern, forming in particular, the bifurcate salt-bridges D778•••H776 and D796••• H776 (Fig 7).

In the native KIT, the salt-bridge D792•••R815 is equivalent to that found in CSF-1R^WT^. In addition, D810 is bound to R791 (R791•••D810), completing a pattern of strong and paired salt-bridge interactions stabilizing the KIT binding pocket, which is smaller than that in CSF-1R (Fig 7). In KIT mutants, the salt-bridge pattern is considerably changed. In KIT^D816V^ mutant, the most resistant to Imatinib, the salt-bridge D792•••R815 is absent. In KIT^V560G^, sensitive to Imatinib, residue D792 interacts either with R791 or with R815. In addition, in this mutant we observed the salt-bridge interaction D810•••K623 found in CSF-1R^WT^. The Imatinib complex formed by mutant KIT^S628N^ shows salt-bridges pattern similar to KIT^WT^, nevertheless the interactions stability is different, reflected in a lower occurrence when compared to the native target.

Such change of the salt-bridges pattern in the mutants compared to the native proteins is prompted by an alternation of the atomic fluctuations (measured by the RMSFs) observed principally in molecular fragments containing the residues involved in the salt-bridges formation. Our results suggest that in Imatinib•target complexes formed by the native receptors (CSF-1R^WT^/KIT^WT^) and by their mutants, sensitive (KIT^S628N^) and very sensitive (KIT^V560G^) to Imatinib, the charges of residues localized at proximity of the binding pocket are equilibrated through alternative salt-bridges which apparently contributes to the stabilization of the binding pocket, providing the high affinity of Imatinib. In complexes formed by the resistant mutants, CSF-1R^D802V^ and KIT^D816V^, the limited and locally positioned salt-bridges favor an excess of positive charges on residues non-involved in salt-bridges at proximity of the binding pocket (Fig D in S1 File) that would interfere with the Imatinib binding.

## Conclusions

The gain-of-function mutations in RTKs alter not only their tight factor-depending regulation but also influence their sensitivity to inhibitors, inducing drug resistance. In particular, it was demonstrated that the V560G substitution in KIT promotes higher sensitivity to Imatinib, while the substitution D816V in KIT and D802V in CSF-1R induces resistance to this drug either systematically (in KIT), or occasionally (in CSF-1R).

Our *in silico* calculation performed on molecular dynamics trajectories demonstrate that the native proteins and their mutants show different binding energy values with Imatinib, correlating with the experimental data. The main factor that drives the targets responsiveness to drug, sensitivity or resistance, is the electrostatic interactions between the protonated inhibitor and the negatively charged residues in the ATP-binding site or in the proximity of this site. The per residue energy decomposition indicates that the Asp to Val substitution in CSF-1R^D802V^ and KIT^D816V^ contributes the most to falling of the binding energy compared to the other KIT mutants. The energy calculations of complexes formed with the KIT targets having either the partially or entirely cleaved JMR showed that the JMR role in the Imatinib binding is accessory. The salt-bridges pattern suggests that in the resistant mutants, CSF-1R^D802V^ and KIT^D816V^, a charge redistribution within residues at the vicinity of the ATP-binding site favors the repulsion of the positively charged Imatinib.

## Supporting Information

S1 File **Scheme A. Structural formula of Imatinib**. Atoms participating in the H-bonding with the targets are shown. **Fig A**. **Imatinib docking (best poses) into the targets, CSF-1R and KIT**. Structural formula of Imatinib is shown in the first box. Imatinib and protein residues that interact directly with Imatinib are represented in sticks and the protein backbone is shown in grey as cartoon. H-bonds between the protein and the ligand are shown as dotted lines. **Fig B**. **MD simulations of the KIT-Imatinib complexes**. (**A**) The Root Mean Square Deviation (RMSD) values of the complexes formed by Imatinib and KIT^WT^ (top) and its mutant KIT^D816V^ (bottom) were calculated for the backbone atoms from replicas 1 and 2 separately (black and red) of MD simulations of the studied systems. (**B**) Root Mean Square Fluctuations (RMSFs) computed on the backbone atoms from replicas 1 and 2 separately (black and red). **Fig C. Binding energy of imatinib•KIT targets**. Graphical representation of the free binding energy (ΔG) of Imatinib•target complexes. The MM-PBSA calculations were performed for KIT^WT^, KIT^S628N^ and KIT^D816V^ containing the partial JMR, identic to that in the mutant KIT^V560G^. The total ΔG energy value is shown for each complex. The ΔG value for KIT^V560G^ is reproduced for comparison. **Fig D**. **Electrostatic potential (EP) surface of the Imatinib•target complexes**. EP surface for each complex was obtained on a representative equilibrated conformation before the MD simulations. EP calculations on the Connolly solvent-accessible surfaces of the receptors were performed with the APBS software. The color scale ranges from red (electronegative potential) through white (neutral) to blue (electropositive potential). **Table A. Imatinib binding to the targets**. Occurrences of the H-bonds between Imatinib and the targets residues were calculated for each of the two MD replicas (1 and 2). Imatinib atoms participating in the interactions with the targets are represented in scheme A in S1 File. The atom pairs for donor-acceptor interactions are depicted in the Table. **Table B. The free energy of binding in the Imatinib-RTK complexes (RTK = KIT**^**WT**^ **and KIT**^**D816V**^**)** The free binding energy (ΔG_bind_) and contributions of electrostatic (ΔG_elec_t), van der Waals (ΔG_vdw_) and solvation (ΔG solv) energies were calculated using MM-PBSA for each of the two MD replicas (1 and 2). All energies are in kcal/mol. **Table C. Per residue contribution to the final binding energy in Imatinib•CSF-1R complexes**. Contribution of all residues from the ATP binding pockets and the point mutation (distinguished by yellow and orange background, respectively), and all residues contributing with ΔΔG > 4 kcal/mol were considered. The favorable and unfavorable contributions of residues are highlighted in green and red colour, respectively. Std is standard deviation. **Table D. Per residue contribution to the final binding energy in Imatinib•KIT complexes**. Contribution of all residues from the ATP binding pockets and the point mutation (distigushed by yellow and by orange background, respectively) and all charged residues contributing with ΔΔG > 4 kcal/mol were considered. The favorable and unfavorable contributions of residues are highlighted in green and red colour, respectively. Std is standard deviation.
(DOCX)
