# Supplementary material for: Insight on Mutation-Induced Resistance from Molecular Dynamics Simulations of the Native and Mutated CSF-1R and KIT
Source: PLoS One. 2016 Jul 28;11(7):e0160165. doi: 10.1371/journal.pone.0160165 (PMC4965071; doi:10.1371/journal.pone.0160165)
Supplement: S1 File — Scheme A. Structural formula of Imatinib. Atoms participating in the H-bonding with the targets are shown. Fig A. Imatinib docking (best poses) into the targets, CSF-1R and KIT. Structural formula of Imatinib is shown in the first box. Imatinib and protein residues that interact directly with Imatinib are represented in sticks and the protein backbone is shown in grey as cartoon. H-bonds between the protein and the ligand are shown as dotted lines. Fig B. MD simulations of the KIT-Imatinib complexes. (A) The Root Mean Square Deviation (RMSD) values of the complexes formed by Imatinib and KITWT (top) and its mutant KITD816V (bottom) were calculated for the backbone atoms from replicas 1 and 2 separately (black and red) of MD simulations of the studied systems. (B) Root Mean Square Fluctuations (RMSFs) computed on the backbone atoms from replicas 1 and 2 separately (black and red). Fig C. Binding energy of imatinib•KIT targets. Graphical representation of the free binding energy (ΔG) of Imatinib•target complexes. The MM-PBSA calculations were performed for KITWT, KITS628N and KITD816V containing the partial JMR, identic to that in the mutant KITV560G. The total ΔG energy value is shown for each complex. The ΔG value for KITV560G is reproduced for comparison. Fig D. Electrostatic potential (EP) surface of the Imatinib•target complexes. EP surface for each complex was obtained on a representative equilibrated conformation before the MD simulations. EP calculations on the Connolly solvent-accessible surfaces of the receptors were performed with the APBS software. The color scale ranges from red (electronegative potential) through white (neutral) to blue (electropositive potential). Table A. Imatinib binding to the targets. Occurrences of the H-bonds between Imatinib and the targets residues were calculated for each of the two MD replicas (1 and 2). Imatinib atoms participating in the interactions with the targets are represented in scheme A in S1 File. The atom pairs for [file pone.0160165.s001.docx]

**Supporting Information S1**

**Insight on** **Mutation-Induced Resistance from Molecular Dynamics Simulations of the Native and Mutated CSF-1R and KIT**

Priscila da Silva Figueiredo Celestino Gomes, Isaure Chauvot De Beauchêne, Nicolas Panel,

Sophie Lopez, Paulo De Sepulveda, Pedro Geraldo Pascutti, Eric Solary and Luba Tchertanov

**1 Scheme**

**4 Figures**

**4 Tables**

**Scheme A**. **Structural formula of Imatinib**. Atoms participating in the H-bonding with the targets are shown.

**Fig A**. **Imatinib docking (best poses) into the targets, CSF-1R and KIT**. Structural formula of Imatinib is shown in the first box. Imatinib and protein residues that interact directly with Imatinib are represented in sticks and the protein backbone is shown in grey as cartoon. H-bonds between the protein and the ligand are shown as dotted lines.


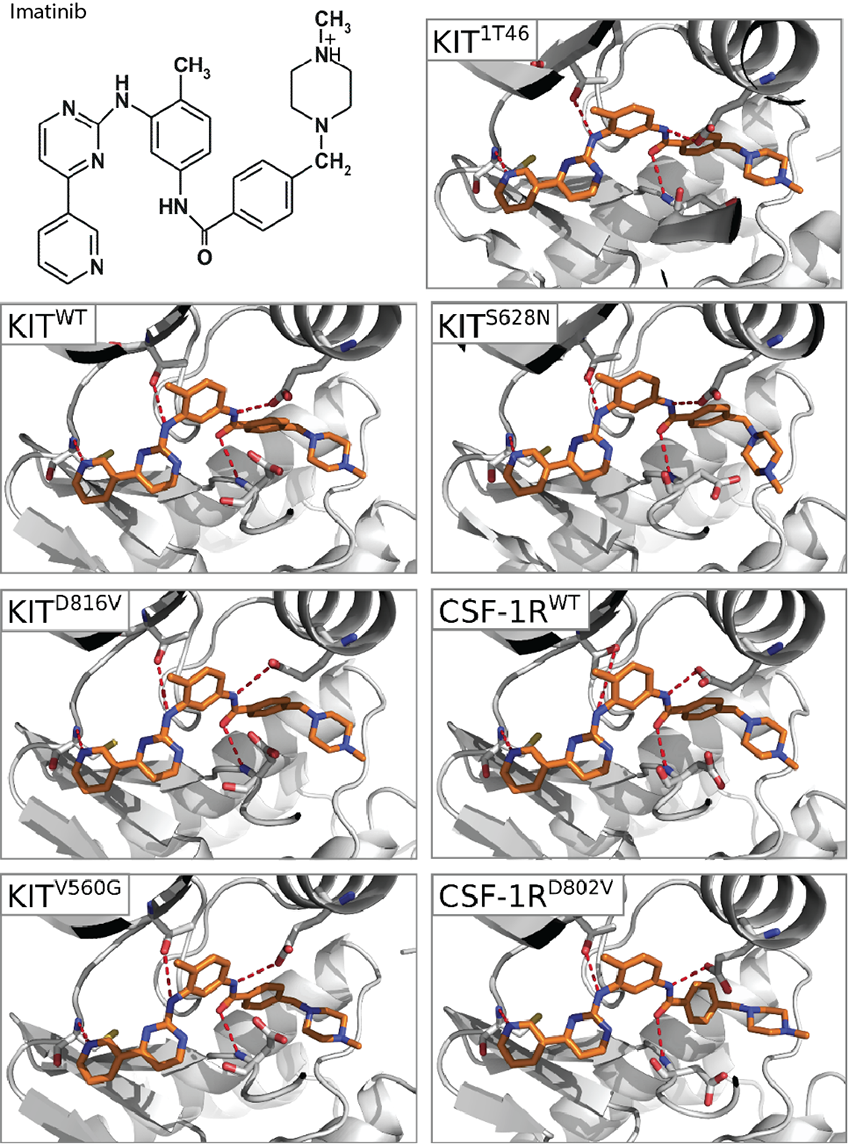


**Fig B**. **MD simulations of KIT-Imatinib complexes.** (**A**) The Root Mean Square Deviation (RMSD) values of the complexes formed by Imatinib and KIT^WT^ (top) and its mutant KIT^D816V^ (bottom) were calculated for the backbone atoms from replicas 1 and 2 separately (black and red) of MD simulations of the studied systems. (**B**) Root Mean Square Fluctuations (RMSFs) computed on the backbone atoms from replicas 1 and 2 separately (black and red).


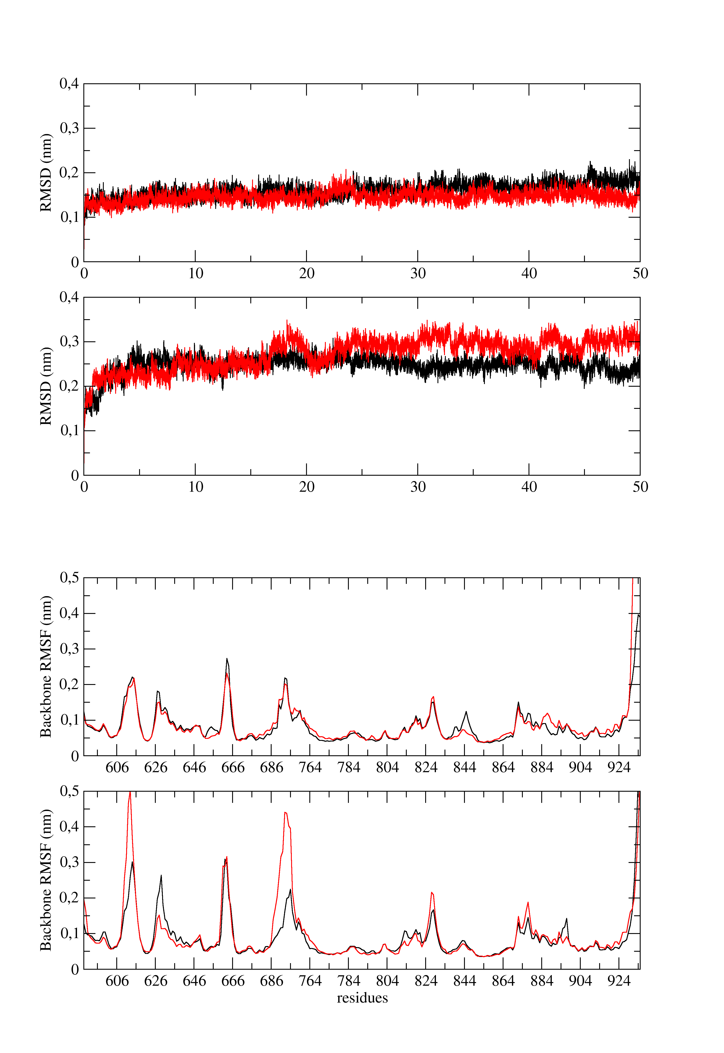


**Fig C.** **Binding energy of imatinib•KIT targets.** Graphical representation of the free binding energy (ΔG) of Imatinib•target complexes. The MM-PBSA calculations were performed for KIT^WT^, KIT^S628N^ and KIT^D816V^ containing the partial JMR, identic to that in the mutant KIT^V560G^. The total ΔG energy value is shown for each complex. The ΔG value for KIT^V560G^ is reproduced for comparison.


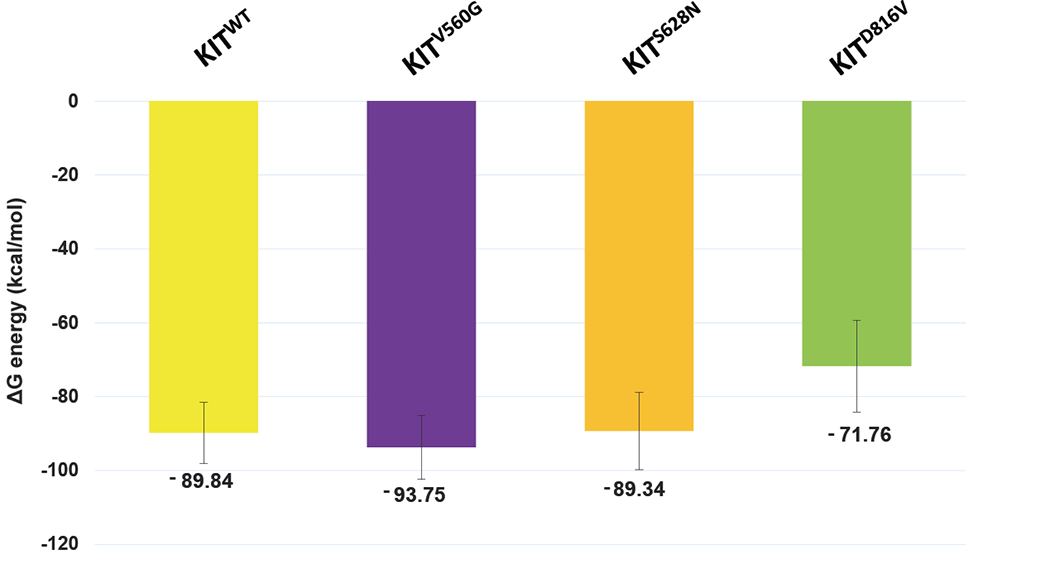


**Fig D**. **Electrostatic potential (EP) surface of the Imatinib•target complexes**. EP surface for each complex was obtained on a representative equilibrated conformation before the MD simulations. EP calculations on the Connolly solvent-accessible surfaces of the receptors were performed with the APBS software. The color scale ranges from red (electronegative potential) through white (neutral) to blue (electropositive potential).


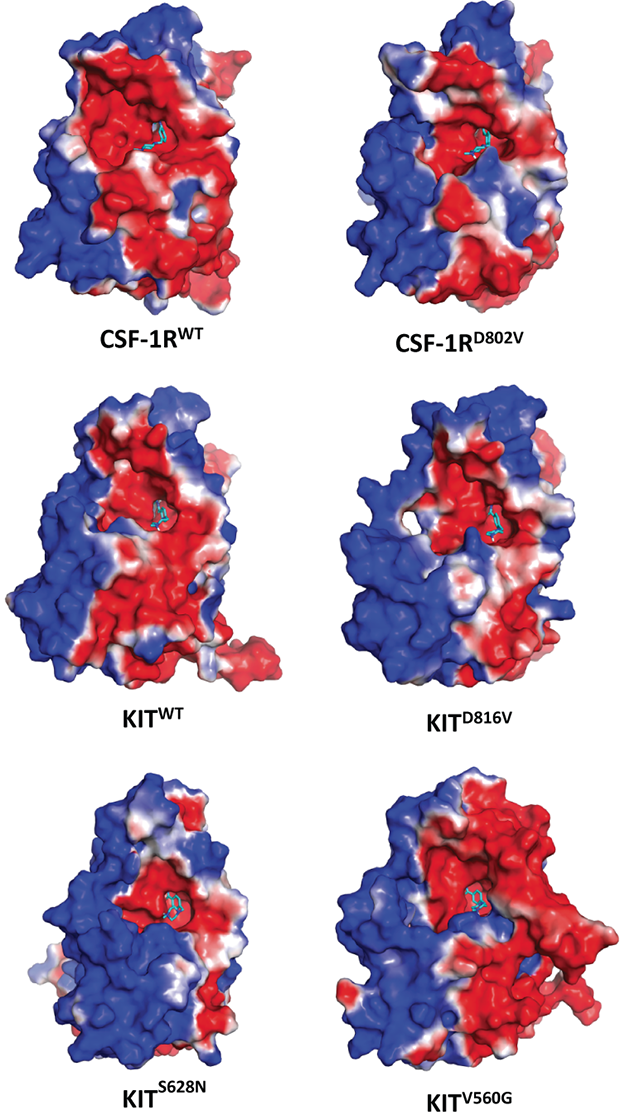


**Table A.** **Imatinib binding to the targets.** Occurrences (%) of the H-bonds between Imatinib and the targets residues were calculated for each of the two MD replicas (1 and 2). Imatinib atoms participating in the interactions with the targets are represented in scheme 1 in S1 file. The atom pairs for donor-acceptor interactions are depicted in the Table.


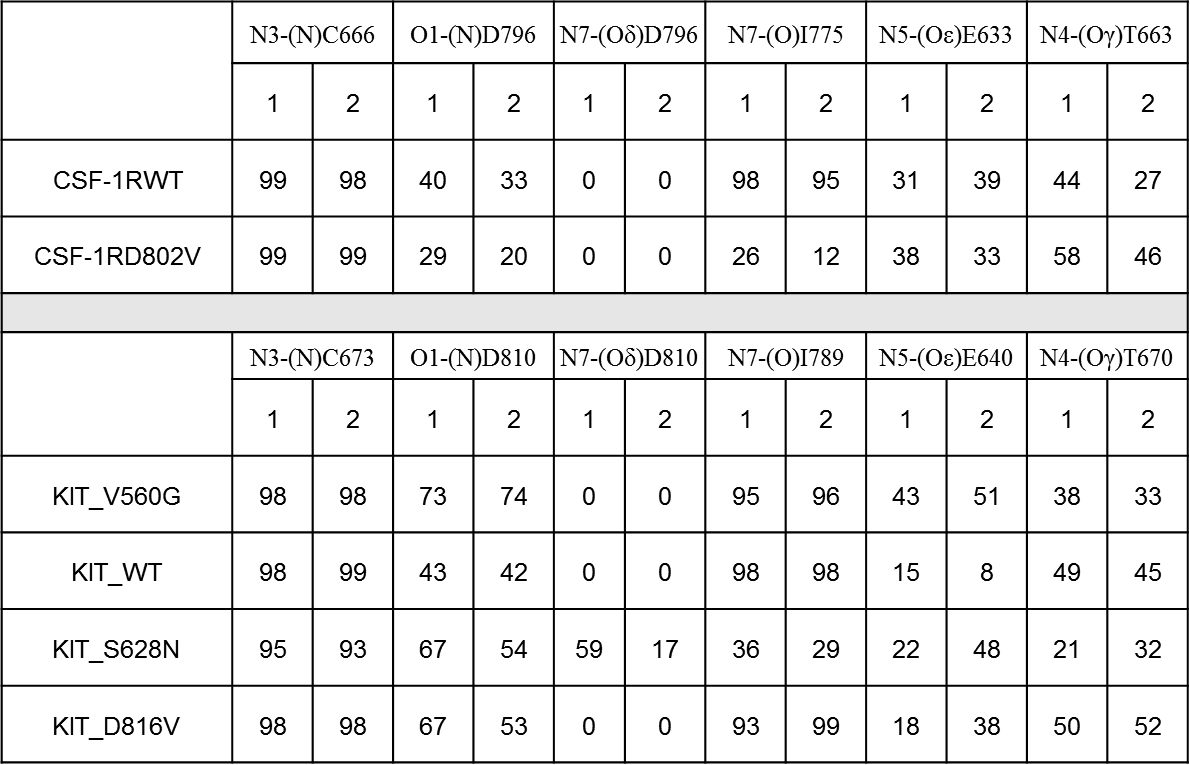


**Table B.** **The free energy of binding in the Imatinib-RTK complexes (RTK = KIT^WT^ and KIT^D816V^)** The free binding energy (ΔG_bind_) and contributions of electrostatic (ΔG_elec_t), van der Waals (ΔG_vdw_) and solvation (ΔG solv) energies were calculated using MM-PBSA for each of the two MD replicas (1 and 2). All energies are in kcal/mol. All numerical values are presented as in output file.

|  | ΔG_vdw_ | | ΔG_elect_ | | ΔG_sol_ | | ΔG_bind_ | |
| --- | --- | --- | --- | --- | --- | --- | --- | --- |
| RTK | 1 | 2 | 1 | 2 | 1 | 2 | 1 | 2 |
| KIT^WT^ | -66.46 ± 3.48 | -67.94 ±3.17 | -70.86 ± 11.95 | -77.29 ± 11.36 | 80.74 ± 5.73 | 81.25 ± 5.60 | -56.58 ± 8.09 | -63.97 ± 7.81 |
| KIT^D816V^ | -65.73 ± 3.41 | -66.38 ± 3.11 | -53.19 ± 10.80 | -59.72 ± 10.10 | 79.04 ± 6.78 | 80.56 ± 5.11 | -39.87 ± 8.01 | -45.54 ± 7.63 |

**Table C.** **Per residue contribution to the final binding energy in Imatinib•CSF-1R complexes**. Contribution of all residues from the ATP binding pockets and the point mutation (distinguished by yellow and orange background, respectively), and all residues contributing with ΔΔG > 4 kcal/mol were considered. The favorable and unfavorable contributions of residues are highlighted in green and red colour, respectively. Std is standard deviation. All numerical values are presented as in output file.

| Residues  type | Sequence number | CSF-1RWT | std | CSF-1RD802V | std |
| --- | --- | --- | --- | --- | --- |
| LEU | 582 | 5.72 | 0.17 | 5.68 | 0.22 |
| LYS | 586 | 6.26 | 0.37 | 6.29 | 0.32 |
| LYS | 595 | 6.91 | 0.26 | 6.95 | 0.36 |
| GLU | 598 | -6.26 | 0.29 | -6.25 | 0.29 |
| LYS | 606 | 4.48 | 0.27 | 4.47 | 0.29 |
| GLU | 607 | -4.53 | 0.26 | -4.53 | 0.28 |
| ASP | 608 | -4.26 | 0.16 | -4.21 | 0.16 |
| LYS | 612 | 5.31 | 0.20 | 5.30 | 0.19 |
| LYS | 616 | 15.95 | 2.47 | 18.29 | 2.01 |
| LYS | 619 | 7.54 | 0.74 | 7.55 | 0.88 |
| ASP | 625 | -10.36 | 0.99 | -9.44 | 1.40 |
| GLU | 626 | -10.57 | 1.73 | -9.23 | 1.45 |
| LYS | 627 | 7.39 | 0.32 | 7.61 | 0.51 |
| GLU | 628 | -11.15 | 0.76 | -11.31 | 1.09 |
| GLU | 633 | -17.73 | 1.76 | -18.73 | 2.10 |
| LYS | 635 | 11.60 | 0.88 | 12.06 | 1.11 |
| THR | 663 | 0.15 | 0.74 | 0.12 | 0.69 |
| GLU | 664 | -5.81 | 0.55 | -5.80 | 0.66 |
| CYS | 666 | -2.26 | 0.48 | -2.30 | 0.45 |
| ASP | 670 | -8.59 | 0.30 | -8.66 | 0.33 |
| ARG | 676 | 6.88 | 0.35 | 7.20 | 0.32 |
| ARG | 677 | 6.52 | 0.27 | 5.61 | 0.24 |
| LYS | 678 | 5.29 | 0.15 | 5.38 | 0.18 |
| ARG | 680 | 5.19 | 0.46 | 5.56 | 0.23 |
| GLU | 683 | -5.87 | 0.37 | -5.05 | 0.20 |
| ASP | 685 | -4.53 | 0.19 | -5.04 | 0.33 |
| ARG | 753 | 4.61 | 0.15 | 4.67 | 0.28 |
| ASP | 754 | -5.32 | 0.12 | -5.33 | 0.21 |
| LYS | 772 | 10.31 | 0.55 | 10.40 | 1.06 |
| ILE | 775 | -4.24 | 0.75 | -2.27 | 1.18 |
| ARG | 777 | 18.60 | 1.93 | 16.11 | 2.05 |
| ASP | 778 | -14.80 | 1.28 | -15.50 | 1.67 |
| ARG | 782 | 9.88 | 0.47 | 10.45 | 1.14 |
| LYS | 793 | 6.95 | 0.43 | 6.89 | 0.64 |
| ASP | 796 | -18.08 | 1.57 | -17.83 | 1.48 |
| ARG | 801 | 11.81 | 1.25 | 10.94 | 0.67 |
| ASP/VAL | 802 | -8.66 | 0.68 | -0.01 | 0.07 |
| ASP | 806 | -8.68 | 0.53 | -9.21 | 0.57 |
| LYS | 812 | 7.64 | 0.73 | 7.48 | 0.57 |
| ARG | 816 | 7.79 | 0.50 | 8.26 | 0.78 |
| LYS | 820 | 6.78 | 0.23 | 7.00 | 0.32 |
| GLU | 825 | -8.78 | 0.20 | -8.15 | 0.35 |
| ASP | 829 | -7.82 | 0.22 | -7.19 | 0.33 |
| ASP | 837 | -15.15 | 0.61 | -13.70 | 1.01 |
| GLU | 847 | -8.11 | 0.25 | -8.09 | 0.31 |
| LYS | 864 | 4.82 | 0.14 | 4.75 | 0.15 |
| LYS | 867 | 5.12 | 0.20 | 5.02 | 0.24 |
| LYS | 870 | 6.38 | 0.36 | 6.14 | 0.36 |
| ASP | 871 | -5.40 | 0.17 | -5.17 | 0.22 |
| LYS | 883 | 4.47 | 0.17 | 4.25 | 0.14 |
| GLU | 896 | -6.45 | 0.31 | -6.02 | 0.35 |
| ARG | 900 | 8.46 | 0.17 | 7.90 | 0.34 |
| GLU | 912 | -5.14 | 0.14 | -4.99 | 0.28 |
| GLU | 916 | -4.36 | 0.14 | -4.37 | 0.23 |
| ASP | 917 | -4.22 | 0.12 | -4.29 | 0.17 |
| ARG | 918 | 4.11 | 0.31 | 3.68 | 0.15 |
| ARG | 919 | 4.44 | 0.17 | 4.59 | 0.16 |
| GLU | 920 | -4.42 | 0.27 | -3.72 | 0.13 |
| ARG | 921 | 4.65 | 0.18 | 4.72 | 0.21 |
| ASP | 922 | -8.83 | 0.29 | -8.80 | 0.31 |

**Table D.** **Per residue contribution to the final binding energy in Imatinib•KIT complexes.** Contribution of all residues from the ATP binding pockets and the point mutation (distigushed by yellow and by orange background, respectively) and all charged residues contributing with ΔΔG > 4 kcal/mol were considered. The favorable and unfavorable contributions of residues are highlighted in green and red colour, respectively. Std is standard deviation. All numerical values are presented as in output file.

| Residue type | Sequence number | KITWT | std | KITV560G | std | KITS628N | std | KITD816V | std |
| --- | --- | --- | --- | --- | --- | --- | --- | --- | --- |
| GLY* | 560 |  |  | -0.33 | 0.07 |  |  |  |  |
| GLU* | 561 |  |  | -9.20 | 0.47 |  |  |  |  |
| GLU* | 562 |  |  | -8.25 | 0.60 |  |  |  |  |
| ASP* | 572 |  |  | -12.60 | 1.06 |  |  |  |  |
| ASP* | 579 |  |  | -8.69 | 0.49 |  |  |  |  |
| LYS* | 581 |  |  | 7.94 | 0.42 |  |  |  |  |
| GLU* | 583 |  |  | -6.41 | 0.44 |  |  |  |  |
| ARG* | 586 |  |  | 6.69 | 0.16 |  |  |  |  |
| ARG* | 588 |  |  | 4.91 | 0.16 |  |  |  |  |
| LEU | 589 | 5.65 | 0.18 | 0.05 | 0.02 | 5.88 | 0.23 | 5.67 | 0.17 |
| LYS | 593 | 6.21 | 0.26 | 6.17 | 0.28 | 6.37 | 0.34 | 6.20 | 0.26 |
| LYS | 602 | 6.92 | 0.21 | 6.94 | 0.21 | 7.09 | 0.25 | 6.90 | 0.23 |
| GLU | 605 | -6.21 | 0.23 | -6.21 | 0.24 | -6.31 | 0.25 | -6.22 | 0.21 |
| ASP | 615 | -4.15 | 0.16 | -4.21 | 0.15 | -4.28 | 0.18 | -4.21 | 0.21 |
| LYS | 623 | 15.22 | 1.64 | 17.36 | 1.69 | 15.71 | 1.84 | 17.54 | 1.79 |
| LYS | 626 | 7.37 | 0.38 | 7.72 | 0.49 | 7.67 | 0.43 | 7.27 | 0.56 |
| SER/ASN | 628 | -0.07 | 0.06 | -0.09 | 0.07 | -0.07 | 0.10 | -0.01 | 0.11 |
| GLU | 633 | -10.78 | 0.93 | -12.17 | 1.83 | -10.69 | 0.99 | -10.92 | 1.02 |
| ARG | 634 | 7.64 | 0.37 | 7.49 | 0.28 | 7.45 | 0.36 | 7.73 | 0.37 |
| GLU | 635 | -11.14 | 0.90 | -10.87 | 0.75 | -10.97 | 1.13 | -11.05 | 0.81 |
| GLU | 640 | -16.69 | 1.65 | -18.73 | 1.48 | -18.19 | 1.95 | -17.57 | 1.73 |
| LYS | 642 | 11.77 | 1.07 | 10.70 | 0.48 | 11.73 | 0.99 | 11.79 | 1.03 |
| THR | 670 | 0.15 | 0.70 | 0.41 | 0.70 | 0.48 | 0.79 | 0.18 | 0.69 |
| GLU | 671 | -5.94 | 0.50 | -6.08 | 0.47 | -5.93 | 0.52 | -6.07 | 0.44 |
| CYS | 673 | -2.33 | 0.41 | -2.22 | 0.41 | -2.09 | 0.61 | -2.24 | 0.40 |
| ASP | 677 | -8.36 | 0.42 | -8.28 | 0.30 | -8.68 | 0.46 | -8.58 | 0.28 |
| ARG | 683 | 6.88 | 0.30 | 6.76 | 0.29 | 7.09 | 0.29 | 6.90 | 0.35 |
| ARG | 684 | 5.74 | 0.16 | 5.75 | 0.19 | 5.94 | 0.28 | 6.02 | 0.28 |
| LYS | 685 | 5.18 | 0.10 | 5.19 | 0.11 | 5.26 | 0.14 | 5.17 | 0.11 |
| ARG | 686 | 5.35 | 0.20 | 5.43 | 0.24 | 5.56 | 0.27 | 5.47 | 0.32 |
| ASP | 687 | -4.73 | 0.13 | -4.76 | 0.12 | -4.83 | 0.15 | -4.88 | 0.27 |
| LYS | 693 | 3.57 | 0.15 | 4.25 | 0.23 | 3.57 | 0.16 | 3.75 | 0.17 |
| GLU | 758 | -5.32 | 0.21 | -5.28 | 0.27 | -5.39 | 0.31 | -4.84 | 0.38 |
| ASP | 759 | -5.48 | 0.32 | -5.38 | 0.33 | -5.19 | 0.49 | -5.39 | 0.28 |
| ASP | 760 | -5.18 | 0.11 | -5.19 | 0.11 | -5.25 | 0.13 | -5.18 | 0.10 |
| GLU | 761 | -4.25 | 0.11 | -4.23 | 0.10 | -4.26 | 0.11 | -4.23 | 0.11 |
| ASP | 765 | -4.73 | 0.09 | -4.77 | 0.09 | -4.75 | 0.10 | -4.77 | 0.08 |
| GLU | 767 | -4.92 | 0.12 | -4.99 | 0.13 | -4.91 | 0.14 | -4.96 | 0.13 |
| ASP | 768 | -5.39 | 0.10 | -5.44 | 0.10 | -5.40 | 0.12 | -5.43 | 0.10 |
| LYS | 778 | 6.83 | 0.26 | 7.01 | 0.29 | 6.71 | 0.25 | 6.90 | 0.30 |
| LYS | 786 | 10.06 | 0.42 | 9.92 | 0.39 | 9.93 | 0.41 | 10.16 | 0.43 |
| ILE | 789 | -4.28 | 0.64 | -4.40 | 0.77 | -2.43 | 1.27 | -4.31 | 0.74 |
| ARG | 791 | 19.31 | 1.84 | 18.63 | 1.60 | 21.19 | 1.50 | 18.12 | 1.63 |
| ASP | 792 | -14.54 | 1.23 | -16.48 | 1.26 | -13.69 | 1.19 | -14.23 | 0.83 |
| ARG | 796 | 9.55 | 0.37 | 9.18 | 0.41 | 9.91 | 0.53 | 9.83 | 0.33 |
| ARG | 804 | 5.24 | 0.10 | 5.29 | 0.10 | 5.29 | 0.14 | 5.30 | 0.10 |
| LYS | 807 | 6.84 | 0.41 | 7.12 | 0.37 | 7.01 | 0.41 | 7.10 | 0.36 |
| ASP | 810 | -19.94 | 1.35 | -18.25 | 1.28 | -21.28 | 3.10 | -18.78 | 1.31 |
| ARG | 815 | 13.47 | 0.83 | 10.43 | 0.71 | 11.97 | 1.16 | 11.56 | 1.10 |
| ASP/VAL | 816 | -8.86 | 0.32 | -8.58 | 0.42 | -9.36 | 0.52 | -0.17 | 0.10 |
| LYS | 818 | 8.22 | 0.56 | 8.14 | 0.52 | 8.51 | 0.72 | 7.18 | 0.51 |
| ASP | 820 | -8.88 | 0.36 | -8.58 | 0.37 | -9.26 | 0.47 | -9.25 | 0.40 |
| LYS | 826 | 6.99 | 0.34 | 7.26 | 0.57 | 6.98 | 0.35 | 7.28 | 0.70 |
| ARG | 830 | 8.15 | 0.45 | 7.68 | 0.44 | 8.31 | 0.43 | 7.89 | 0.57 |
| LYS | 834 | 6.92 | 0.20 | 6.83 | 0.20 | 7.07 | 0.25 | 7.17 | 0.30 |
| GLU | 839 | -9.05 | 0.33 | -9.14 | 0.22 | -8.19 | 0.37 | -9.18 | 0.23 |
| GLU | 849 | -10.28 | 0.29 | -10.24 | 0.25 | -9.77 | 0.51 | -10.25 | 0.26 |
| ASP | 851 | -15.32 | 0.64 | -15.35 | 0.48 | -14.55 | 0.74 | -15.53 | 0.56 |
| GLU | 861 | -8.01 | 0.22 | -8.01 | 0.21 | -7.96 | 0.26 | -8.08 | 0.19 |
| ASP | 876 | -5.63 | 0.15 | -6.03 | 0.27 | -5.99 | 0.34 | -6.19 | 0.25 |
| LYS | 881 | 5.00 | 0.14 | 4.94 | 0.13 | 5.02 | 0.22 | 4.94 | 0.13 |
| LYS | 884 | 6.20 | 0.33 | 6.11 | 0.28 | 6.09 | 0.36 | 6.11 | 0.33 |
| GLU | 885 | -5.16 | 0.16 | -5.01 | 0.11 | -5.01 | 0.15 | -5.03 | 0.12 |
| ARG | 888 | 6.28 | 0.27 | 6.48 | 0.11 | 6.07 | 0.28 | 6.49 | 0.12 |
| GLU | 893 | -4.78 | 0.24 | -4.76 | 0.20 | -4.73 | 0.20 | -4.75 | 0.21 |
| GLU | 898 | -5.03 | 0.13 | -5.12 | 0.17 | -4.98 | 0.15 | -5.09 | 0.13 |
| ASP | 901 | -5.49 | 0.16 | -5.54 | 0.15 | -5.40 | 0.16 | -5.45 | 0.15 |
| LYS | 904 | 5.21 | 0.13 | 5.26 | 0.12 | 5.14 | 0.12 | 5.22 | 0.12 |
| ASP | 908 | -6.47 | 0.13 | -6.52 | 0.11 | -6.26 | 0.18 | -6.50 | 0.12 |
| ASP | 910 | -6.55 | 0.26 | -6.54 | 0.22 | -6.22 | 0.28 | -6.58 | 0.24 |
| LYS | 913 | 6.02 | 0.15 | 6.05 | 0.13 | 5.80 | 0.19 | 6.03 | 0.14 |
| ARG | 914 | 8.56 | 0.22 | 8.61 | 0.15 | 8.01 | 0.30 | 8.63 | 0.17 |
| LYS | 918 | 7.96 | 0.64 | 8.22 | 0.61 | 7.77 | 0.61 | 7.90 | 0.62 |
| GLU | 925 | -5.95 | 0.19 | -5.98 | 0.19 | -5.83 | 0.21 | -5.86 | 0.17 |
| LYS | 926 | 5.12 | 0.21 | 5.22 | 0.21 | 5.01 | 0.23 | 5.04 | 0.14 |
| GLU | 930 | -4.36 | 0.21 | -4.50 | 0.42 | -4.51 | 0.15 | -4.60 | 0.13 |
| ILE | 935 | -4.68 | 0.82 | -5.19 | 0.90 | -4.10 | 0.53 | -3.99 | 0.32 |
